# Rational design framework for fluorescent biosensors from periplasmic binding proteins

**DOI:** 10.1042/BSR20260131

**Published:** 2026-05-08

**Authors:** Martín González-Andrade, Abril Gijsbers, Alejandro Sosa-Peinado, Nathaly Vasquez-Martínez

**Affiliations:** Departamento de Bioquímica, Facultad de Medicina, Universidad Nacional Autónoma de México, Ciudad de México 04510, México

**Keywords:** fluorescent biosensor, LAO, mBBr, periplasmic binding protein, rational design, site-specific labeling

## Abstract

Periplasmic binding proteins (PBPs) are attractive scaffolds for fluorescent biosensors because they undergo large ligand-induced conformational changes and exhibit high specificity. Despite their potential, generalizable design strategies have been hindered by an incomplete understanding of how labeling positions influence protein dynamics and signal transduction. Here, we established a systematic experimental framework using the lysine-arginine-ornithine binding protein (LAO) as a model to characterize PBP-based fluorescent biosensors. Seven positions classified as endosteric, peristeric, or allosteric relative to the binding site were labeled with monobromobimane (mBBr). Our characterization revealed position-dependent effects on quantum yields, fluorescence intensity changes, ligand-binding affinities, and thermal stability. Molecular dynamics simulations (MD) of wild-type and mBBr-labeled variants provided a mechanistic context for these experimental observations. Functional biosensor positions (D51C, D53C, K228C, Y230C, and E167C) maintained open-like conformations necessary for signal generation upon ligand encounter. In contrast, peristeric position A89C, located at the hinge region, spontaneously adopted a closed-like conformation even in the absence of ligand, resulting in an inverse fluorescence response and reduced affinity. These results demonstrate that while structural criteria can guide initial site selection, experimental validation remains essential, as static structures alone cannot predict the complex interactions among labeling position, dynamics, and biosensor function. The experimental and computational framework established here with LAO as a model system provides methodological principles that may inform biosensor engineering from other PBP scaffolds, while emphasizing that conformational dynamics must be considered alongside static structural criteria.

## Introduction

Biosensors are analytical devices designed to detect the presence or measure the concentration of specific target molecules, comprising two fundamental components: a receptor that binds the target and a transducer that converts receptor binding into a detectable signal. These systems range from integrated devices that combine biological receptors with external physical transducers to molecular sensors where the biological molecule itself transduces binding events into measurable outputs [1]. Over recent years, biosensors have experienced significant development driven by applications in healthcare diagnostics, drug discovery, environmental monitoring, and disease biomarker detection, owing to their sensitivity, selectivity, and real-time monitoring capabilities [2–5]. Despite their considerable biotechnological potential, it remains difficult to establish general design strategies for biosensors. This limitation has three interconnected causes. First, there are no universal methods to generate receptors for diverse molecular targets. Second, strategies to transduce binding into signal outputs are insufficient. Third, it is difficult to achieve tunable dynamic ranges for different applications [6].

Periplasmic binding proteins (PBPs) are attractive scaffolds for engineering fluorescent biosensors due to their large ligand-induced conformational changes, high specificity, and affinities ranging from nanomolar to micromolar. These proteins share a conserved two-domain architecture connected by a hinge region, with a ligand-binding site located in the interdomain cleft. Upon ligand binding, PBPs undergo a transition from an “open” (apo) to a “closed” (ligand-bound) conformation, which can be transduced into optical signals through site-specific labeling with environment-sensitive fluorophores [7–10]. This conformational switch has enabled engineering of various PBP scaffolds into functional biosensors. These include sensors based on maltose-binding protein (MBP), glucose-binding protein, ribose-binding protein, xylose-binding protein, and amino acid-binding proteins, as well as phosphate- and metal-ion-binding proteins, demonstrating their versatility for *in vitro* and *in vivo* applications [8,11–15]. Recent advances have extended this approach to include computational strategies for rational fluorophore site selection, highlighting the growing role of molecular dynamics simulations (MD) in guiding PBP-based biosensor design before experimental validation [15]. Together, these developments position PBP-based fluorescent biosensors as a versatile platform within the broader field of genetically encoded fluorescent biosensors for real-time monitoring of cellular signals and metabolites [16].

**Figure 1:**
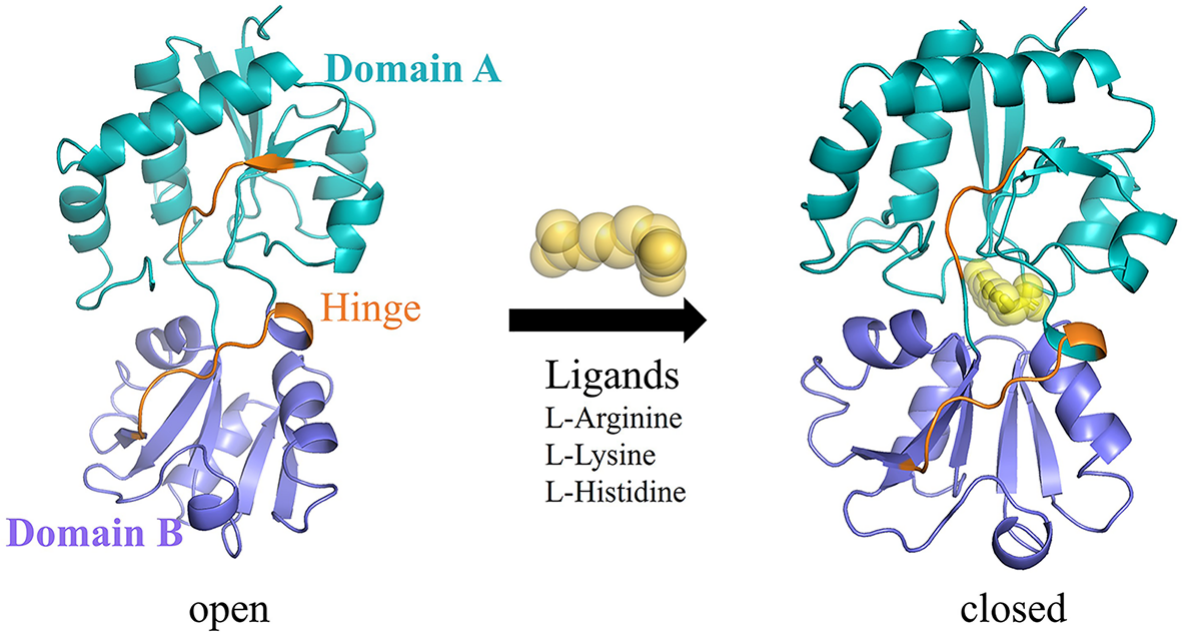
Conformational states of LAO Representative crystal structures showing LAO in the open conformation (PDB: 2LAO) and closed conformation (PDB: 1LAF) bound to L-arginine (yellow). The arrow indicates ligand-induced closure of the binding cleft.

The lysine-arginine-ornithine binding protein (LAO) from *Salmonella typhimurium* is a well-suited model system for establishing biosensor design principles. LAO is a 238-residue, two-domain PBP with residues 1–88 and 195–238 forming domain A, and residues 93–185 forming domain B. Well-characterized crystal structures exist for both open and closed conformations bound to L-arginine, L-lysine, and L-histidine (Figure 1) [17,18]. Upon ligand binding, the protein undergoes a substantial reorientation of domain B, exhibiting affinities in the nanomolar range (*K*_d_ = 14–29 nM) [19]. Ten residues form the ligand-binding site at the interface between the two domains (Tyr-14, Phe-52, Asp-30, Ser-69, Ser-70, Ser-72, Arg-77, Leu-117, Thr-121, and Asp-161). Although LAO is not suitable for direct biosensor applications because it functions in bacterial transport systems and has limited applicability in eukaryotic cells, it has well-defined conformational states and high-resolution structures. These features make it a useful platform for developing biosensor design strategies that can be transferred to other PBPs.

The present study describes an experimental methodology for characterizing fluorescent biosensors derived from PBPs. Using LAO as a model system, we developed a library of fluorescent LAO variants through site-specific labeling with monobromobimane (mBBr), an environment-sensitive fluorophore whose emission properties are modulated by solvent accessibility and local polarity [20]. We selected positions based on structural criteria derived from crystal structures, prioritizing spatial distribution relative to the binding site, location within α-helical regions, and solvent accessibility in both open and closed conformations. Seven positions spanning endosteric, peristeric, or allosteric sites were successfully labeled and characterized with respect to spectroscopic properties, ligand-binding affinities, and thermal stability. MD in four systems—apo, unliganded closed conformation LAO, L-arginine-bound, and each of the seven mBBr-labeled single-cysteine variants in the apo conformation—provided mechanistic insight into the conformational dynamics underlying biosensor function [15,21]. The integration of experimental characterization and MD applied here to LAO may inform biosensor engineering strategies from other PBP scaffolds, underscoring that static structural metrics alone are insufficient to predict sensor performance.

## Materials and methods

### Construction of wild-type and single-cysteine mutant LAO

The *argT* gene encoding LAO protein was amplified from *S. typhimurium* genomic DNA using forward (5′-GGGAATTCCATATGAAGAAGACCGTTCTCGC-3′) and reverse (5′-CGCGGATCCGGCGCGTTATCCCCTTCCCTC-3′) primers containing *Nde*I and *Bam*HI sites, respectively. The PCR product was digested with *Nde*I and *Bam*HI, purified using the QIAquick Gel Extraction Kit (Qiagen, 28704), cloned into pGEM-T Easy Vector (Promega Corporation, A1360), and subsequently subcloned into the pET12b expression vector (MilliporeSigma, 69433), using the same restriction sites. The resulting constructs were used to transform competent *Escherichia coli* DH5α cells (Thermo Scientific, EC0112).

Seven single-cysteine mutants of LAO were generated at positions D51, D53, R131, E167, A89, K228, and Y230 by site-directed mutagenesis using the pET12b-LAO wild-type plasmid as a template. Mutagenesis reactions were carried out using the QuikChange Site-Directed Mutagenesis Kit (Agilent Technologies, 200518) with Pfu DNA polymerase according to the manufacturer’s protocol, using the mutagenic primers listed in Table 1. PCR amplification was conducted with 100 ng of template plasmid and 125 ng of each primer under the following conditions: initial denaturation at 95°C for 1 min, followed by 25 cycles of denaturation at 95°C for 1 min, annealing at 55°C for 1 min, and extension at 68°C for 10 min, with a final extension at 68°C for 10 min. After amplification, PCR products were digested with *Dpn*I at 37°C for 1 h to remove parental methylated DNA, and the resulting fragments were used to transform competent *E. coli* DH5α cells. All constructs were verified by DNA sequencing using an ABI PRISM 310 Genetic Analyzer (Thermo Fisher Scientific) at the Instituto de Fisiología Celular, UNAM, México.

**Table 1:** Sequences of mutagenic primers

Mutants	Forward primer	Reverse primer
**D51C**	GGGTCGCCAGCTGCTTTGATGCGC	GCGCATCAAAGCAGCTGGCGACCC
**D53C**	CCAGCGACTTTTGTGCGCTTATTCCCT	AGGGAATAAGCGCACAAAAGTCGCTGG
**A89C**	GACAAACTTTACTGTGCGGATTCACG	CTGTTTGAAATGACACGCCTAAGTG
**R131C**	GATAACTGGTGCACTAAAGGTG	CACCTTTAGTGCACCAGTTATC
**E167C**	GTCGCCGCCAGCTGTGGTTTCCTGAA	CAGCGGCGGTCGACACCAAAGGACTT
**K228C**	TTACGACAAAATGGCCTGCAAGTACTTCGA	CATTAAAATCGAAGTACTTGCAGGCCATTTTG
**Y230C**	GGCCAAAAAGTGCTTCGATTTTAATG	CATTAAAATCGAAGCACTTTTTGGCC

### Recombinant expression and purification of LAO variants

The pET12b-LAO wild-type and mutant plasmids were used to transform *E. coli* BL21(DE3) cells. Transformed cells were cultured in LB medium containing 100 μg/ml ampicillin at 37°C with shaking at 250 rpm. Protein expression was induced with 0.45 mM isopropyl β-D-1-thiogalactopyranoside (IPTG, Sigma–Aldrich, I6758) over 4 h at 37°C. Cells were harvested by centrifugation at 3800 × ***g*** for 10 min, and periplasmic proteins were extracted by osmotic shock. Briefly, the cell pellets were suspended in buffer containing 10 mM potassium acetate (pH 5.1), 20% (w/v) sucrose, and 1 mM EDTA and incubated at room temperature for 10 min. Following centrifugation at 3800 × ***g*** for 15 min, pellets were suspended in 5 mM MgSO_4_ and gently stirred on ice for 10 min. The supernatant containing the periplasmic LAO protein was collected after final centrifugation at 11 800 × ***g*** for 10 min at 4°C.

The supernatants were purified using a two-step chromatography protocol. Cation-exchange chromatography was performed on a Source 15S column (Cytiva, 17518201) equilibrated with 10 mM potassium acetate buffer, pH 5.1, with LAO eluting at 70 mM NaCl in a linear gradient from 0 to 200 mM over 20 min. Subsequently, anion-exchange chromatography was performed using a Source 15Q column (Cytiva, 17518101) equilibrated with 5 mM bis-Tris propane buffer, pH 8.5, to separate ligand-bound (closed conformation) LAO from ligand-free (open conformation) LAO. The two conformational forms eluted at 37.5 and 47.5 mM NaCl, respectively, in a linear gradient from 0 to 100 mM over 40 min. For single-cysteine mutants, 5 mM 2-mercaptoethanol was maintained throughout purification to prevent intermolecular disulfide bond formation. Protein purity was monitored by sodium dodecyl sulfate–polyacrylamide gel electrophoresis (SDS–PAGE). Protein concentrations of wild-type and mBBr-modified mutants were determined using the bicinchoninic acid assay (molar absorptivity of bimane at 380 nm, ε_380_ = 5000 M^-1^ cm^-1^).

### Chemical modification with mBBr

Cysteine mutants of LAO were chemically modified with a 10-fold molar excess of mBBr (Sigma–Aldrich, B4380) [20] in 20% (v/v) DMSO to keep the final organic solvent concentrations below 5% (v/v). Before labeling, protein samples (5–10 mg/ml) were reduced with 20 mM DTT for 24 h at 4°C and buffer-exchanged using a PD-10 Desalting Column (Cytiva, 28989333). The labeling reaction proceeded for 1 h at 25°C in the dark with gentle agitation. After modification, we removed excess fluorophore by size exclusion chromatography using a Superdex 75 column (Cytiva, 17517401). Labeling efficiency was determined by quantifying unreacted cysteine thiols using Ellman’s reagent (5,5′-dithiobis-2-nitrobenzoic acid, DTNB). The reaction product, 5-thio-2-nitrobenzoic acid (TNB), was quantified spectrophotometrically using its molar absorptivity (ε_409_ = 14 150 M^−1^ cm^−1^) at 25°C and pH 7.4 [22].

### Steady-state fluorescence measurements

All fluorescence measurements were performed on a PC1 spectrofluorometer (ISS Inc.) at 30°C using a TC 125 Peltier temperature controller (Quantum Northwest). For wild-type LAO, tryptophan fluorescence was monitored at λ_ex_ = 295 nm with emission collected from 300 to 340 nm. For mBBr-labeled LAO, fluorescence was monitored at λ_ex_ = 381 nm with emission collected from 400 to 550 nm (1 nm intervals, 10 integrations per point). Excitation and emission slit widths were 0.5 and 2 mm, respectively. Protein concentrations were maintained at 1 μM for all experiments.

#### Quantum yield determination

Quantum yields (Φ) of free and protein-bound fluorophores were determined using the comparative method as described by (eqn 1):

(1) $$\phi_{x}=\phi_{est}\frac{F_{x}DO_{est}}{F_{est}DO_{x}}$$

where subscripts *x* and *est* refer to the sample and standard, respectively; Φ is the quantum yield, *F* is the integrated fluorescence intensity over the entire emission spectrum, and OD is the optical density at the excitation wavelength [23]. Quinine sulfate in 1 N H_2_SO_4_ (Φ = 0.55) was used as the fluorescence standard [24]. All quantum yields were determined in the apo (ligand-free) state before any ligand addition, using 1 μM protein in 50 mM Tris–HCl buffer, pH 7.5, at 30°C. Buffer fluorescence was subtracted from all measurements before determining quantum yield.

#### Ligand binding to LAO

Equilibrium fluorescence titrations were performed at 30°C with 1 μM protein in 2 ml of protein dissolved in 50 mM Tris–HCl buffer, pH 7.5. Ligands were added sequentially from concentrated stock solutions (100 mM L-arginine, 100 mM L-lysine, and 310 mM L-histidine) in 1 μl aliquots. Final ligand concentrations ranged from 50 μM to 1 mM for L-arginine and L-lysine, and from 155 μM to 3.1 mM for L-histidine. Fluorescence was recorded after equilibration at each concentration, with total added volume not exceeding 1% of the initial volume. For both wild-type LAO and mBBr-labeled variants, *K*_d_ values were determined by fitting the fluorescence changes as a function of ligand concentration to (eqn 2), which describes a 1:1 binding model [25]:

(2) $$v_{0}=\frac{(1+k_{d}/P_{0}+L_{0}/P_{0})-\sqrt{{\left(1+k_{d}/P_{0}+L_{0}/P_{0}\right)}^{2}-4L_{0}/P_{0}}}{2}$$

where *P*_o_ and *L*_o_ are the total concentrations of protein and ligand, respectively, and *K*_d_ is the dissociation constant.

### Thermal stability

Thermal denaturation of wild-type LAO and mBBr-labeled mutants was monitored by changes in the intrinsic protein fluorescence as a function of temperature. A protein stock solution (1 μM) in 50 mM Tris–HCl buffer, pH 7.5, was placed in a cuvette with constant stirring. Fluorescence was measured at λ*_ex_* = 295 nm and λ*_em_* = 326 nm (slit widths: 0.5 mm excitation and 2 mm emission). Thermal denaturation was monitored from 40 to 70°C at a heating rate of 20°C/h using a TC 125 Peltier temperature controller (Quantum Northwest).

Thermodynamic stability data were analyzed assuming a two-state model (native folded state ⇌ fully unfolded state) at equilibrium. The melting temperature (*T*_m_) and thermodynamic parameters (Δ*H*_D_ and Δ*S*_D_) were determined by fitting the fluorescence data to (eqn 3) [26]:

(3) $$F=\frac{F_{0N}+S_{N}T+(F_{0D}+S_{D}T)\times e^{\left(-\Delta H_{D}^{\circ }+T\Delta S_{D}^{\circ }/RT\right)}}{1+e^{\left(-\Delta H_{D}^{\circ }+T\Delta S_{D}^{\circ }/RT\right)}}$$

where *F*_0N_ and *F_0D_* are the fluorescence intensities of the native and denatured states, respectively; *S*_N_ and *S*_D_ represent the slopes of the pre- and post-transition baselines; Δ*H_D_*° and Δ*S_D_*° are the standard enthalpy and entropy changes for the two-state unfolding reaction; *T* is the temperature in Kelvin, and *R* is the universal gas constant.

The change in free energy of unfolding (ΔΔ*G*) for each mBBr-labeled LAO mutant relative to wild-type was calculated using the approximation of Becktel and Schellman (eqn 4) [27]:

(4) $$\Delta \Delta G=\Delta T_{m}\times \Delta S_{wt}$$

where Δ*T*_m_ is the difference in melting temperature between the labeled mutant and wild-type LAO, and Δ*S*_wt_ is the entropy change between native and unfolded states of wild-type LAO. As this approximation assumes a constant unfolding entropy term across variants, the resulting ΔΔ*G* values should be interpreted as qualitative estimates of relative stability.

### Solvent-accessible surface area calculations

Solvent-accessible surface area (SASA) was calculated using the Lee–Richards algorithm as implemented in NACCESS V2.1.1 [28]. SASA values were determined from crystal structures in the open conformation (PDB: 2LAO) and closed conformations complexed with different ligands: L-arginine (PDB: 1LAF), L-lysine (PDB: 1LAO), L-ornithine (PDB: 1LAO), and L-histidine (PDB: 1LAH). SASA calculations were performed for individual residues to identify positions with significant solvent exposure and conformational changes (ΔSASA) between open and closed states. Additionally, SASA values of the mBBr moiety were calculated from structural models of mBBr-labeled mutants to predict fluorophore solvent exposure in different conformational states.

### Structural modeling of mBBr-labeled mutants

Structural models of seven LAO single-cysteine mutants (D51, D53, A89, R131, E167, K228, and Y230) chemically modified with mBBr were generated using HyperChem 7.5 software (HiperCube Inc.). Models were generated based on crystal structures of unliganded LAO and ligand-bound LAO. Selected residues were first mutated to L-cysteine (PDB: 1LST), followed by geometric optimization of the mutation site and energy minimization *in vacuo* using the AMBER force field [29]. The mBBr fluorophore was then covalently attached to each of the thiol groups of the cysteine mutant and subjected to geometric optimization until reaching a minimum root-mean-square deviation (RMSD) gradient of 0.01 kcal/[Å mol]. Crystallographic structures and structural models were visualized using PyMOL version 2.5.0 (Schrödinger, Inc.).

To assess potential fluorescence quenching by neighboring aromatic residues, Cβ–Cβ distances between each labeling position and the tryptophan (W47, W130) and tyrosine (Y14, Y73, Y126, Y142, Y218) residues of LAO were calculated from the open conformation crystal structure (PDB: 2LAO) using a custom Python script. Residues within 15 Å of the labeling position were considered capable of quenching mBBr fluorescence through the tryptophan/tyrosine-induced quenching (TrIQ) mechanism. Results were visualized and verified in PyMOL.

### Molecular dynamics simulation

The coordinates of the crystallographic structures 2LAO, 1LAF, and 1LAF with the arginine ligand were used as starting points for the simulations. Additionally, to investigate the structural and dynamic effects of mBBr labeling at specific positions, seven single-cysteine mutants with covalently attached mBBr were simulated. The mBBr-cysteine conjugate (CMB residue) was parameterized as a single modified residue using the Antechamber module from AmberTools22 [30].

All systems were prepared using the LEaP module in AmberTools22 with the protein.ff19SB force field [31], incorporating the custom CMB residue library at each mBBr-labeled position. Each system was neutralized with Na^+^ counterions and solvated in an octahedral box with TIP3P water molecules, maintaining a minimum wall distance of 12 Å. Covalent bonds involving hydrogen atoms were constrained by applying the SHAKE algorithm, which allowed the use of a 2-fs time step to integrate Newton’s equations according to the Amber package recommendations [30,32]. Systems underwent energy minimization with protein restraints (2500 steps) followed by unrestrained minimization (5000 steps). The systems were then gradually heated to 298.15 K over 50 ps under constant volume with restraints on the protein, using a Langevin thermostat [33]. Equilibration continued at constant pressure (1 atm) with a Monte Carlo barostat, first with protein restraints (100 ps) and then without restraints (500 ps) [34]. Following equilibration, independent 200-ns production MDs were performed. For the three reference systems (apo LAO, unliganded closed LAO, and L-arginine-bound LAO), three independent replicas were run. For the seven mBBr-labeled single-cysteine variants, a single 200-ns trajectory was performed per system.

The calculations were performed using the GPU-accelerated pmemd.cuda engine in AMBER [35] on an Ubuntu 22.04.5 workstation equipped with an Nvidia Gigabyte GeForce RTX 4090 GPU. Trajectory analysis was performed using the CPPTRAJ module from AmberTools22 [30,36]. RMSD and root-mean-square fluctuation (RMSF) were calculated for backbone atoms (C, Cα, N) relative to the equilibrated structures. Radius of gyration (Rg) and SASA were calculated using all heavy atoms and all residues, respectively. The results were plotted and analyzed using OriginLab version 9.0 (OriginLab Corporation). For comparative analysis, the wild-type apo LAO trajectories served as the conformational reference against which all mBBr-labeled variants were assessed. To comprehensively assess the flexibility of LAO in complex with the ligands, the MDLovoFit package (Institute of Chemistry, University of Campinas) was employed [37]. VMD [38] and PyMOL were used to visualize and create MD images.

## Results and discussion

### Expression, purification, and conformational separation of LAO variants

**Figure 2:**
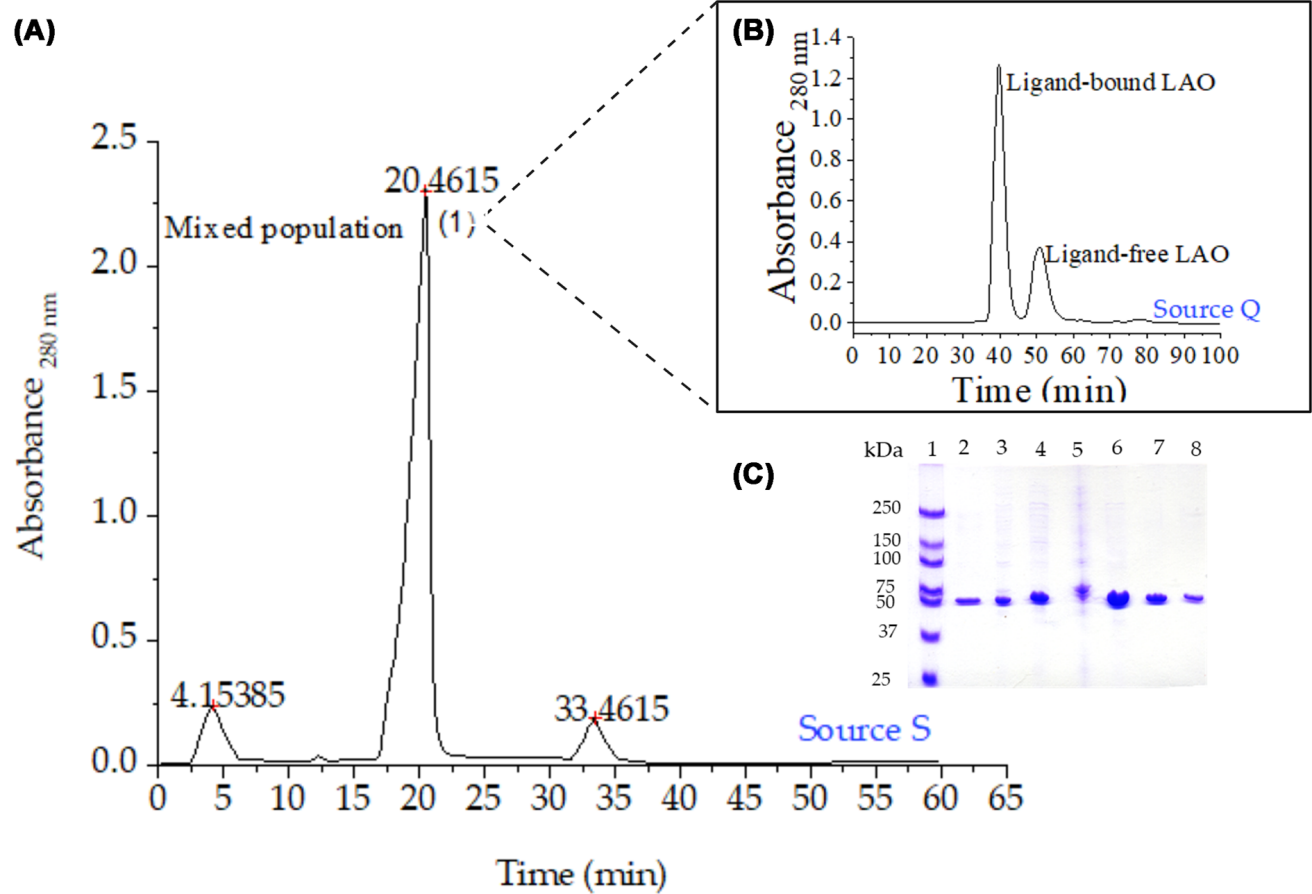
LAO protein purification by sequential ion-exchange chromatography (**A**) In the first purification step, LAO protein was eluted as a single, highly concentrated peak (1) with 70 mM NaCl using cation-exchange chromatography (Source S, pH 5.1). (**B**) In the second purification step, ligand-bound LAO (37 mM NaCl) was separated from free LAO (47.5 mM NaCl) by anion-exchange chromatography (Source Q, pH 8.5). (**C**) SDS–PAGE shows the fractions collected during the purification process. Lanes: 1, molecular weight marker; 2, culture medium; 3, periplasmic extract after sucrose treatment; 4, periplasmic extract after MgSO_4_ treatment; 5, cell pellet after osmotic shock; 6, pooled fractions from Superdex 75 size-exclusion chromatography; 7, purified LAO from Source S cation-exchange chromatography; 8, ligand-bound LAO from Source Q anion-exchange chromatography. Periplasmic extracts are enriched for periplasmic proteins due to the selective osmotic shock method, resulting in fewer bands compared with whole-cell lysates.

Wild-type LAO and seven single-cysteine variants were successfully expressed and purified using a three-step protocol: osmotic shock extraction, followed by cation-exchange and anion-exchange chromatography, yielding approximately 20 mg/l of protein per liter of culture. Anion-exchange chromatography at pH 8.5 resolved two distinct conformational populations: ligand-bound and ligand-free forms. This assignment was supported by fluorescence titration. The early-eluting fraction showed a negligible response to L-arginine addition, consistent with a ligand-occupied binding site, while the late-eluting fraction showed a 39% fluorescence increase upon ligand saturation, consistent with its identity as ligand-free LAO. Both conformational states showed high purity as assessed by SDS–PAGE (Figure 2).

### Structural basis for mutant selection

Structural analysis of LAO identified key functional regions that guided the design of the mutants. The seven cysteine variants were selected by simultaneously applying four structural criteria: (i) spatial classification relative to the binding site to establish generalizable design principles across all three categories; (ii) preferential location within α-helical secondary structure to minimize structural destabilization [20]; (iii) SASA ≥40 Å^2^ in the open (apo) conformation to avoid excessive protein perturbation; and (iv) substantial ΔSASA between open and closed conformations, averaged over four crystallographic structures with different bound ligands.

Based on SASA calculations and spatial distribution, seven positions were selected for single-cysteine substitution: D51C, D53C (endosteric); A89C, R131C, E167C (peristeric); and K228C, Y230C (allosteric) (Supplementary Figure S1, Table 2). Six positions met the SASA ≥40 Å^2^ threshold in the open conformation and showed substantial ΔSASA values between open and closed conformations, ranging from 11.68 to 64.04 Å^2^. Position A89C, with SASA = 14.63 Å^2^, was included as an exception due to its strategic location at the interdomain hinge, providing an opportunity to test whether positions with low initial solvent exposure could function as biosensors.

**Table 2:** SASA values of residues selected for cysteine substitution in LAO

Variants	LAO (Å^2^)	LAO- L-Arg (Å^2^)	LAO- L-Lys (Å^2^)	LAO- L-Orn (Å^2^)	LAO- L-His (Å^2^)	ΔSASA (Å^2^)^*^
D51C	109.64	70.59	72.95	72.50	72.57	39.05
D53C	127.39	63.29	66.68	59.88	63.54	64.04
R131C	117.35	150.65	149.27	144.09	147.78	30.57
A89C	14.63	34.28	33.68	36.92	36.78	19.65
E167C	50.68	27.71	27.14	31.41	31.42	22.97
K228C	153.25	73.25	74.25	76.77	74.05	78.67
Y230C	42.53	54.21	52.69	52.65	51.93	11.68

All SASA values were computationally determined using the Lee–Richards algorithm (NACCESS v2.1.1) from PDB-deposited crystal structures. They do not represent direct experimental measurements.

* ΔSASA = SASA (open) – SASA (closed), averaged over four ligand-bound crystal structures (L-arginine, L-lysine, L-ornithine, L-histidine).

### Characterization of wild-type LAO

#### Spectroscopic characterization

**Figure 3:**
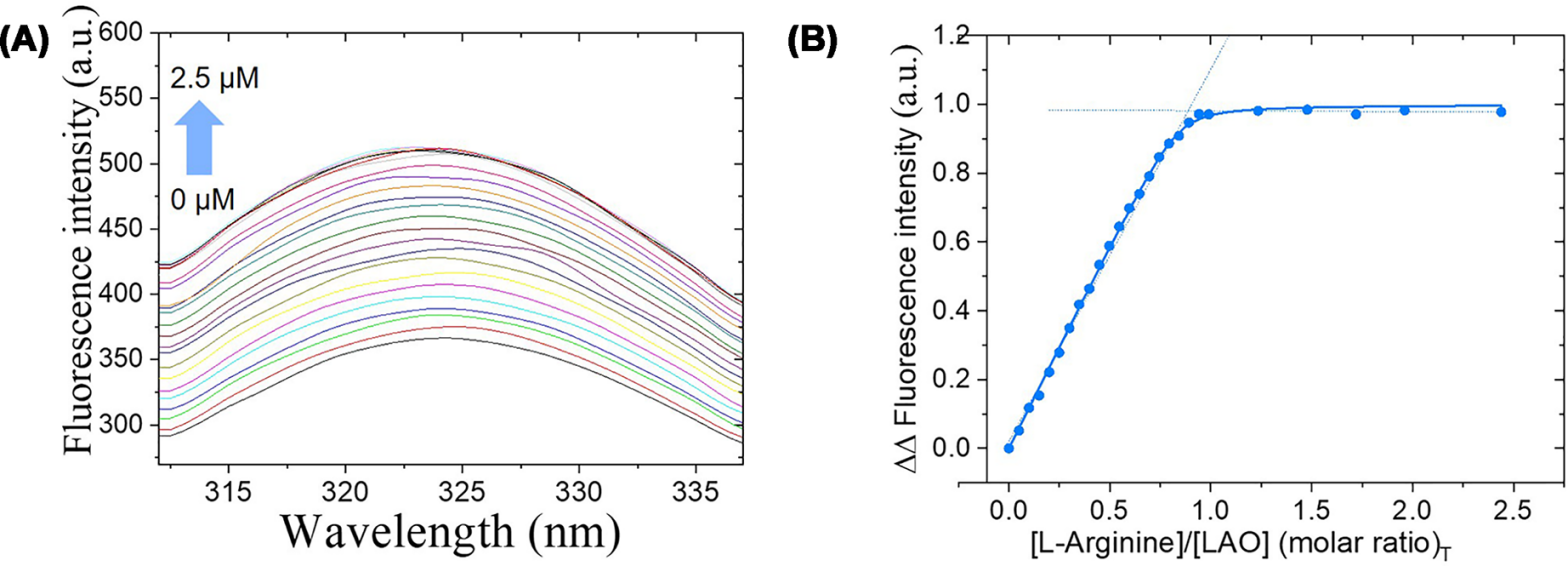
Ligand binding of wild-type LAO (**A**) Fluorescence titration of apo-LAO (1 μM) with L-arginine. The colored lines represent the progressive increase in tryptophan fluorescence (λ_em_ = 324 nm) as ligand concentration increases from 0 to 2.5 μM (indicated by the arrow) upon ligand binding. (**B**) Binding isotherm fit to 1:1 model (*K*_d_ = 6 ± 0.1 nM). Fluorescence intensity is expressed in arbitrary units (a.u.).

Both ligand-free and ligand-bound LAO showed absorption maxima at 278 nm, but differences in UV-visible absorption and fluorescence emission indicated successful separation (Figure 3A and Supplementary Figure S2). Functional separation was further supported by titration with L-arginine. Ligand-free LAO had a 39% fluorescence increase upon ligand saturation, whereas ligand-bound LAO showed negligible fluorescence change, enabling accurate determination of binding affinities.

#### Ligand-binding affinity

Equilibrium fluorescence titrations were performed by monitoring the increase in the intrinsic fluorescence intensity of the proteins at 324 nm upon ligand binding. This fluorescence enhancement reflects changes in the local environment of tryptophan residues (W47 and W130) during the conformational transition from the open to the closed state. L-arginine and L-lysine bound with high affinity (*K*_d_ = 6 and 8 nM, respectively) with 1:1 binding stoichiometries (0.97 and 1.1 ligand:protein, respectively). Figure 3 shows representative data for L-arginine; L-lysine showed equivalent behavior (Supplementary Figure S3). In contrast, L-histidine showed substantially weaker binding (*K*_d_ = 687 nM) with 1:1 stoichiometry, reflecting weaker electrostatic interactions between the imidazole side chain and binding site residues optimized for longer, positively charged side chains (Supplementary Figure S3) [18,19]. These binding properties are consistent with the known ligand selectivity of LAO, supporting its use as a model system for biosensor development.

#### pH independence

**Figure 4:**
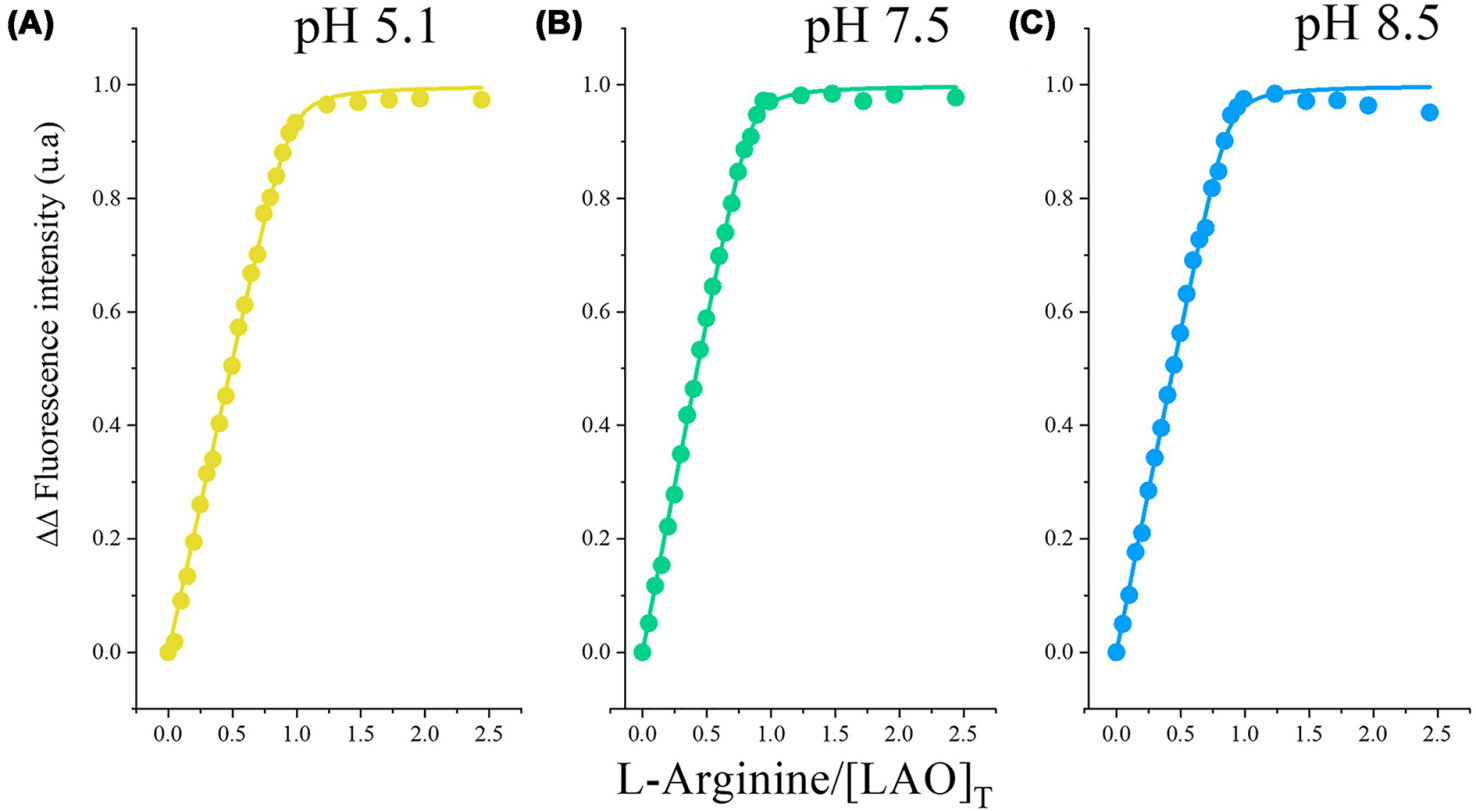
pH dependence of L-arginine binding to wild-type LAO Fluorescence titrations of LAO (1 μM) with **(A)** L-arginine at pH 5.1 (10 mM potassium acetate), **(B)** pH 7.5 (50 mM Tris–HCl), and **(C)** pH 8.5 (5 mM bis-Tris propane) at 30°C. Solid lines represent fits to a 1:1 binding model. Binding affinity remains consistent across the pH range.

To assess the pH dependence of ligand binding, we performed fluorescence titrations with L-arginine and L-lysine at pH levels 5.1, 7.5, and 8.5 (Figure 4). L-arginine maintained consistent affinity across all pH values tested (*K*_d_ = 8 nM at pH 5.1; 6 nM at pH 7.5 and 8.5). L-lysine showed nanomolar affinity at physiological pH (*K*_d_ = 8–18 nM at pH 7.5–8.5) but exhibited ∼6-fold weaker binding at pH 5.1 (*K*_d_ = 46 nM), likely reflecting altered protonation of binding site residues (Asp-30, Asp-161). The guanidinium group of L-arginine (pKa ∼12.5) remains fully protonated across the pH range tested. In contrast, the amino group of L-lysine (pKa ∼10.5) and acidic binding site residues are more sensitive to pH changes, explaining the differential pH dependence. The binding affinities measured across the pH range tested (5.1–8.5) reflect conditions relevant to distinct biological contexts, including acidic environments such as the gastric mucosa, lysosomes, and tumor microenvironments; physiological conditions in most tissues and biofluids; and the bacterial periplasm, the native environment of LAO. This is advantageous as it ensures consistent performance across diverse biological environments [10,39,40].

### Characterization of mBBr-labeled mutants

After characterizing wild-type LAO, we labeled the seven single-cysteine mutants with mBBr to evaluate their potential as fluorescent biosensors. Labeling efficiency was assessed using DTNB, which indicated near-complete labeling (>95%) for all seven variants with minimal residual free thiols detected. This high efficiency, achieved through a 10-fold molar excess of mBBr and overnight incubation, ensured that subsequent fluorescence, binding, and thermostability measurements reflected the properties of homogeneously labeled protein populations.

#### Spectroscopic characterization

**Figure 5:**
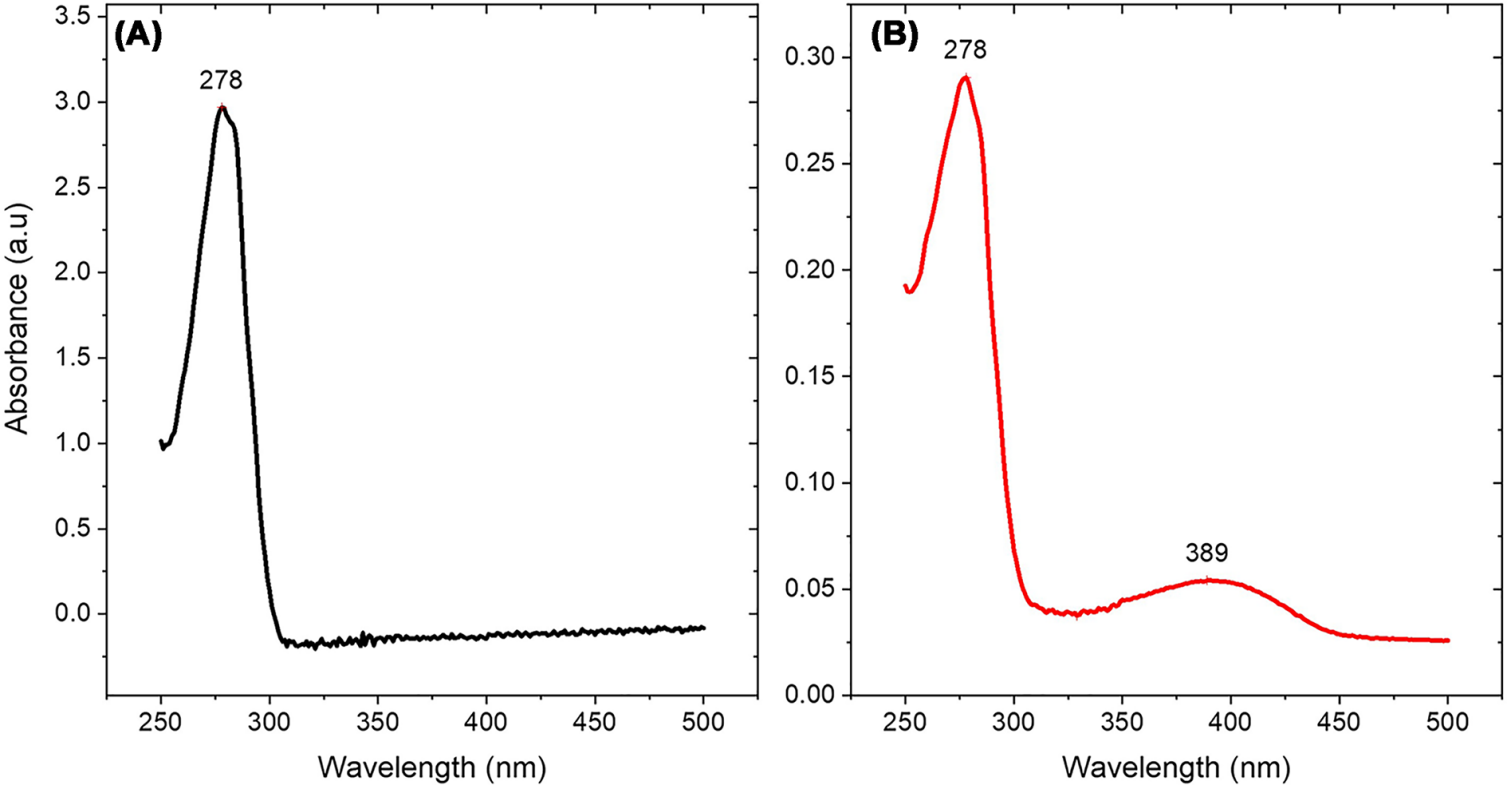
UV-visible absorption spectra confirming mBBr labeling (**A**) Unlabeled LAO. (**B**) mBBr-labeled LAO. E167C variant, shown as a representative example due to its highest quantum yield.

UV-visible absorption spectra were consistent with successful mBBr labeling. Unlabeled LAO showed only the expected protein absorption peak at 278 nm (intrinsic protein fluorophores) (Figure 5A). In contrast, mBBr-labeled mutants showed this protein peak plus an additional peak at 389 nm characteristic of mBBr (Figure 5B). All seven positions were successfully labeled mBBr-labeled; mutants displayed emission maxima between 468 and 475 nm with minimal wavelength shifts (≤8 nm) upon ligand saturation (Table 3).

**Table 3:** Spectral and affinity parameters of site-specifically mBBr-labeled LAO mutants

Variant	λ_max_, Apo (nm)	λ_max_, Sat (nm)	ΔF(%)^a^	φ^b^	*K*_d_ (nM)^c, d^		
					L-Arg	L-Lys	L-His
Wild type^c^	324	324	+ 39%	0.033	6 ± 0.10	8 ± 0.21	687 ± 3.68
D51C	473	472	+ 12%	0.239	7 ± 0.30	54 ± 1.36	257 ± 9.30
D53C	473	472	+ 13%	0.101	7 ± 0.24	10 ± 0.97	95 ± 3.45
A89C	471	475	− 17%	0.043	16 ± 0.38	66 ± 0.55	897 ± 12.71
R131C^c^	325	324	+ 26%	0.024	2 ± 0.89	25 ± 1.11	715 ± 9.53
E167C	468	473	+ 30%	0.773	66 ± 3.68	143 ± 3.36	5663 ± 99.71
K228C	473	475	+ 21%	0.057	4 ± 0.31	8 ± 0.62	320 ± 3.52
Y230C	474	471	+ 34%	0.012	20 ± 0.41	98 ± 2.15	4049 ± 34.41

λmax, ΔF, and φ were determined by steady-state fluorescence spectroscopy.

Reported values are mean ± standard deviation from n ≥ 3 independent experiments.

a Percent change in fluorescence intensity upon ligand saturation relative to apo state

b Quantum yields (φ) were determined in the apo (ligand-free) conformation

c *K*_d_ values were obtained from equilibrium fluorescence titrations fitted to a 1:1 binding model (eqn 2).

d Values for wild-type LAO and R131C were measured by intrinsic tryptophan fluorescence (λ_ex = 295 nm, λ_em = 324 nm). All other variants were characterized using bimane fluorescence (λ_ex = 381 nm, λ_em = 468–475 nm).

#### Position-dependent biosensor properties

The seven mBBr-labeled positions exhibited diverse properties depending on their spatial location relative to the ligand-binding site and interdomain hinge (Tables 2-3). Fluorescence responses upon ligand binding ranged from −17% to +34%, and ligand-binding affinities varied from near-native to 20-fold reduced. Thermal unfolding, monitored by intrinsic fluorescence as a function of temperature, showed reversible two-state transitions for all variants (Supplementary Figure S4, Table 4). While residue SASA (Table 2) guided initial position selection, fluorophore SASA values reported in Table 4 reflect the degree of mBBr burial upon domain closure and were used to interpret the observed fluorescence responses.

**Table 4:** Structural and thermal stability of the mBBr-labeled LAO mutants

Variant	SASA open (Å^2^)	SASA closed (Å^2^)	ΔSASA (Å^2^)^a^	*T_m_* (°C)^b^	ΔΔ*G* (kcal.mol^−1^)^c^
D51C	264	224	-40	54.4	3.00
D53C	291	188	-103	57.2	1.55
A89C	116	133	+17	53.6	3.42
R131C	203	209	+6	54.7	2.85
E167C	180	166	−14	55.6	2.38
K228C	265	272	+7	52.3	4.09
Y230C	197	165	−32	49.7	5.44

a ΔSASA calculated as SASA (open) – SASA (closed); negative values indicate mBBr burial upon domain closure.

b *T_m_* of wild-type LAO = 60.2°C.

c ΔΔ*G* = |Δ*T_m_* | × Δ*S*_WT_, where Δ*S*_WT_ = + 0.518 kcal·mol^−1^·K^−1^ is the entropy of denaturation of wild-type LAO, calculated as Δ*H_D (_*_WT)_/ *T_m (_*_WT)_. Δ*T_m_* = *T_m (_*_WT)_ − *T_m_* (variant).

Wild-type LAO had a *T*_m_ of 60.2°C, whereas labeled mutants ranged from 49.7 to 57.2°C. Despite these *T*_m_ reductions, estimated changes in unfolding free energy were moderate to significant (ΔΔ*G* = 1.55 to 5.44 kcal/mol), producing measurable destabilization relative to wild type, with allosteric positions showing comparatively greater destabilization than endosteric ones. These values should be interpreted cautiously, as the Becktel–Schellman approximation assumes a constant unfolding entropy term that may not hold across variants with altered conformational dynamics [41–43].

Ligand-binding affinities and fluorescence responses of mBBr-labeled variants were assessed by fluorescence titration with L-arginine (Supplementary Figure S5). Endosteric positions D51C and D53C maintained near-native binding affinities for all three ligands despite proximity to the ligand-binding site. Both showed moderate increases in fluorescence, correlating with substantial mBBr burial and moderate thermal destabilization. The combination of maintained affinity, robust fluorescence response, and structural stability suggests both positions as promising biosensor candidates (Tables 3 and 4).

Allosteric positions K228C and Y230C, distant from both the binding site and hinge, showed contrasting properties. K228C showed near-native affinities and a moderate fluorescence response despite increased solvent exposure, suggesting that local microenvironment changes rather than burial drive its signal. Y230C combined the lowest quantum yield with the highest fluorescence response and greatest thermal destabilization yet preserved near-native affinities for L-arginine and L-lysine. Its substantial mBBr burial in Y230C correlated with its large fluorescence change, suggesting that allosteric positions can tolerate local structural perturbations without compromising binding functions (Tables 3 and 4). However, the practical utility of Y230C as a biosensor involves an inherent trade-off between dynamic range and absolute brightness. Although its large fluorescence response is advantageous for detection sensitivity, the very low quantum yield and reduced thermal stability may limit its performance in applications requiring high signal intensity or operation under physiologically demanding conditions and should therefore be carefully considered when selecting labeling positions for specific biosensor applications.

Peristeric positions at or near the interdomain hinge showed the greatest functional variability. E167C achieved the highest quantum yield and robust fluorescence response with moderate mBBr burial, but its ligand affinity was ∼10 to 20-fold lower. This position is therefore sensitive to the effects of substitution on interdomain communication. R131C showed enhanced L-arginine affinity, possibly reflecting elimination of electrostatic repulsion between the positively charged guanidinium group of R131 and the incoming ligand. Unlike other variants, R131C could not be characterized using mBBr fluorescence due to severe TrIQ quenching by nearby aromatic residues (see “Influence of local hydrophobicity and aromatic residue quenching”).

On the other hand, A89C illustrates a case that underscores the importance of hinge-proximal positions. This position was deliberately included despite its low SASA (14.63 Å^2^ in the open state) to test whether positions at the interdomain hinge with minimal solvent exposure could function as biosensors. Unlike all other variants, A89C had an inverse fluorescence response upon ligand binding, accompanied by increased mBBr solvent exposure and reduced affinity for all three ligands. Despite a reasonable quantum yield, A89 demonstrated that hinge positions can compromise the conformational switching mechanism required for signal transduction, even when static structural parameters appear favorable.

These results suggest that static structural parameters have limited predictive power for biosensor performance [41]. Although ΔSASA calculations from crystal structures correctly predicted four successful positions (D51C, D53C, E167C, Y230C), they failed to anticipate the positive response of K228C despite increased mBBr exposure and the inverse fluorescence behavior of A89C. Functional biosensors would require not only substantial ΔSASA but also preservation of native conformational dynamics and local photophysical environments that couple domain motion to fluorescence changes.

#### Influence of local hydrophobicity and aromatic residue quenching on quantum yields

The nearly 100-fold variation in quantum yields across positions cannot be attributed solely to ΔSASA, indicating that additional microenvironment factors modulate mBBr emission. Distance analysis between labeling sites and aromatic residues revealed that TrIQ quenching is a major determinant of quantum yield (Supplementary Table S1).

R131C, located only 5.22 Å from W130 and 5.96 Å from Y126, undergoes near-complete quenching, explaining the absence of detectable bimane fluorescence. The absence of the mBBr signal reflects two converging factors: minimal fluorophore ΔSASA, indicating insufficient environmental change upon conformational transition, and proximity-induced TrIQ quenching that persists independently of conformation. Despite this, intrinsic tryptophan fluorescence showed a Δ*F* = +26% increase upon L-arginine saturation, suggesting perturbation of the W47 and W130 microenvironments during domain closure. This indicates that R131C maintains functional conformational dynamics despite the unfavorable environment for mBBr-based sensing.

D51C (14.33 Å from W47, 11.64 Å from Y14) and D53C (13.41 Å from Y14) show moderate quenching consistent with their intermediate quantum yields. A89C, with Y73 at 10.44 Å and Y218 at 13.88 Å, shows reduced quantum yield alongside its inverse fluorescence response. In contrast, E167C, the brightest position—lies more than 21 Å from all aromatic residues except Y14 (10.01 Å), minimizing quenching. K228C and Y230C show low basal quantum yields due to proximity to Y218 (13.82 Å) and Y14 (12.04 Å), respectively. Despite low quantum yield, K228C shows a moderate fluorescence response because increased solvent exposure upon binding partially relieves quenching. Notably, Y230C exhibits the largest fluorescence response because its highly quenched basal state amplifies the relative environmental change upon domain closure.

Together, these results suggest that ΔSASA alone may be insufficient to predict biosensor performance. Cβ–Cβ distance analysis to aromatic residues should be incorporated as a complementary criterion, as TrIQ quenching and local hydrophobicity are equally critical determinants of quantum yield and fluorescence response magnitude.

### Conformational dynamics

**Figure 6:**
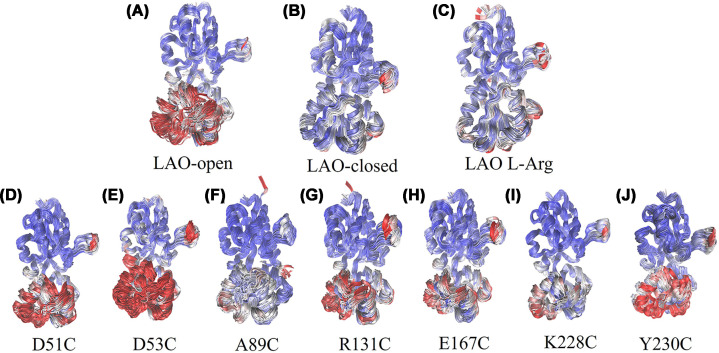
Conformational flexibility from MDLovoFit analysis (**A**) apo-LAO, (**B**) closed apo-LAO, (**C**) L-arginine-bound LAO, and (**D**–**J**) mBBr-labeled variants D51C, D53C, A89C, R131C, E167C, K228C, and Y230C. Residues are colored according to their structural fluctuation calculated by MDLovoFit: the 70% least mobile atoms are shown in blue and the 30% most mobile atoms in red. Visualizations were generated from MDLovoFit output using VMD.

We performed 200-ns MD of wild-type LAO in three states (apo, unliganded closed, and L-arginine-bound) and all seven labeled variants in the apo state. Analysis of RMSD, RMSF, Rg, and SASA provided the mechanistic context for the experimental observations described above. Wild-type LAO exhibited the expected conformational behavior (Figure 6A,B and Supplementary Figures S6–S12). The L-arginine-bound form showed the lowest RMSD and most compact Rg, whereas the apo form had higher RMSD and an elongated structure (Supplementary Figures S6–S12). Simulating L-arginine binding to apo LAO resulted in progressive compaction, reduced solvent exposure, and decreased domain B flexibility, approaching the crystallographic closed state. MDLovofit analysis revealed elevated flexibility in domain B (residues 92–184), particularly the flexible flap at the interdomain hinge, which had the greatest mobility in the apo state (Figure 6C), consistent with previous studies [21].

The labeled variants showed position-dependent effects on stability and dynamics (Figure 6D–J, Supplementary Figures S6–S12). Endosteric mutants reached structural stability, exhibiting RMSD and domain flexibility patterns like the wild-type apo-LAO. D51C showed moderate overall stability and domain B flexibility, like wild-type and D53C (Supplementary Figures S6, S7). D53C exhibited more complex behavior with increasing RMSD, the highest domain B flexibility of all variants, accompanied by progressive Rg compaction approaching closed-like values. This combination of enhanced and global compaction flexibility suggests a dynamic equilibrium between open and partially closed states, which may facilitate the accommodation of smaller ligands, such as L-histidine. Although the 200-ns trajectories are insufficient to capture full domain closure events, which are expected to occur on microsecond-to-millisecond timescales [21], the absence of any closure tendency suggests these variants preserve the conformational landscape required for ligand-induced signal transduction.

In contrast, peristeric mutants showed shifts toward closed-like conformations (Supplementary Figures S8–S10). A89C, at the critical hinge region, behaved differently than expected. Although RMSD and RMSF resembled those of wild-type apo LAO, MDLovofit analysis revealed that A89C adopted a predominantly closed-like state for ∼70% of the simulation, with reduced flexibility in the previously flexible flap region (Figure 6F). This spontaneous closure in the absence of ligand could explain the inverse fluorescence response of A89. If the protein is already closed in the absence of ligand, ligand binding cannot induce further closure and may instead cause local distortions, producing the inverted signal and reduced affinity. R131C and E167C, on the other hand, showed increased regional flexibility compared with wild-type apo LAO (Figure 6G,H), consistent with local conformational perturbations at these peristeric positions. This observation is consistent with the experimentally observed 10–20-fold reduction in ligand-binding affinity, suggesting local binding site distortion rather than global domain rearrangement.

Allosteric mutants maintained a conformational behavior similar to that of wild-type apo LAO, explaining their preserved binding properties (Supplementary Figures S11, S12). K228C adopted a partially closed conformation in the apo state, ranking second among the most compact variants after A89C (Figure 6I). Unlike A89C, however, this partial compaction reflects a local rearrangement at this distal allosteric position that does not occlude the ligand-binding cleft, explaining the preserved near-native affinities. Y230C maintained an open-like conformation despite increased local flexibility at the mutation site (Figure 6J), showing that allosteric positions can tolerate local changes without affecting global conformational dynamics.

These MD results suggest important design considerations. The labeling positions near the interdomain hinge risk shifting the apo-state conformational equilibrium toward closed-like states, abolishing the dynamic range required for signal transduction. Importantly, this conformational pre-organization cannot be predicted from static crystal structures, demonstrating that MD complements experimental characterization by revealing dynamic behaviors invisible to structural snapshots.

#### mBBr fluorophore behavior during molecular dynamics simulations

Because mBBr was modeled explicitly as a CMB in all simulations, it was possible to directly analyze its orientation, solvent accessibility, and interactions with neighboring protein residues throughout the 200-ns trajectories. This analysis complements the assessment of global protein compactness and flexibility and provides a mechanistic interpretation of the quantum yield values and fluorescence responses observed experimentally.

At endosteric positions D51C and D53C, the fluorophore maintained a partially solvent-shielded orientation for most of the simulation, consistent with their moderate quantum yields and positive fluorescence responses. The mBBr solvent accessibility calculated from MD trajectories was consistent with the statically predicted ΔSASA values, supporting the expected fluorophore burial upon domain B closure.

At peristeric position A89C, the fluorophore exhibited persistently high solvent accessibility associated with spontaneous adoption of the closed-like conformation, providing a mechanistic explanation for the inverse fluorescence response. Because the protein is pre-closed, mBBr becomes exposed in a surface cleft rather than undergoing progressive burial upon closure from the open state.

At allosteric position K228C, persistent contacts between mBBr and Y218 were maintained throughout the trajectory. The altered geometry between the fluorophore and Y218 during domain closure contributes to the positive fluorescence response (+21%) despite increased global solvent exposure, demonstrating that partial compaction at distal allosteric positions can be compatible with functional biosensor behavior—in contrast with hinge-proximal pre-closure, which abolishes it. This finding highlights the value of explicitly modeling the fluorophore in simulations, as SASA analysis of the isolated cysteine residue alone does not capture these interactions.

## Conclusions

The present study establishes an experimental framework for characterizing fluorescent biosensors from periplasmic binding proteins using LAO as a model system. Through spectroscopic, thermodynamic, and MD analysis of seven mBBr-labeled variants, we show that structural criteria can provide a useful starting point for position selection. However, experimental validation remains indispensable for identifying functional biosensors. Static structural parameters alone could not predict biosensor performance, as evidenced by positions with favorable SASA predictions that failed as biosensors (A89C) and positions with maintained function despite local structural changes (Y230C).

MD revealed how different positions affected the conformational equilibrium and the flexibility of domain B. The best biosensor positions (endosteric D51C, D53C, and allosteric K228C, Y230C) maintained open-like conformations during simulations, thereby preserving the conformational range required for ligand-induced signal transduction. In contrast, peristeric positions near the interdomain hinge, particularly A89C (included despite low-SASA due to its strategic location at the interdomain hinge), spontaneously shifted toward closed-like conformations, which explained its inverted fluorescence response and reduced binding affinity. These findings suggest that labeling positions should not only report conformational changes but also preserve the native conformational landscape, a requirement that may not be assessed from crystal structures alone and requires both simulations and experimental testing.

The methodology described here addresses a key challenge in biosensor development: predicting which positions will yield functional sensors. Based on our results with LAO as a model system, we propose several design considerations that may inform biosensor development from other PBP scaffolds. First, selecting positions away from the binding site and hinge may minimize functional disruption while maintaining robust signal responses. Second, positions at or near the hinge, particularly those affecting the flexible flap region, should be approached with caution, as they can prevent the conformational switching required for signal transduction. Third, although computational tools are increasingly useful for rational design by identifying potentially non-functional positions, our results emphasize that the diversity of PBP structures and dynamics requires integrating structural analysis, dynamic simulations, and experimental validation for developing functional biosensors for each specific scaffold. Translating these design principles into biosensors suitable for widespread application will further require identifying PBP scaffolds with specificity for biotechnologically or clinically relevant targets and adapting the labeling strategy to each application context.

In this regard, the CMB residue parametrization developed here constitutes a reusable resource that enables prospective evaluation of fluorophore behavior, including solvent accessibility, local interactions, and effects on conformational dynamics, before synthesis and experimental validation. Such a computational-first approach could substantially reduce the number of variants that need to be produced and characterized experimentally, potentially accelerating the biosensor design cycle for other PBP scaffolds.

## Supplementary Material

Supplementary Figures S1-S12 and Table S1

## Data Availability

Supplementary Figures S1–S4 include a structural overview of LAO and mBBr-labeling sites; UV-visible absorption spectra of wild-type LAO; reversible thermal unfolding and refolding assays; and comprehensive MD parameters (RMSD, RMSF, Rg, and SASA) comparing wild-type protein with mBBr-labeled variants (PDF).

## Open Access

Open access for this article was enabled by the participation of Universidad Nacional Autónoma de México in an all-inclusive Read & Publish agreement with Portland Press and the Biochemical Society.

## Abbreviations

LAO: Lysine-arginine-ornithine binding protein
MBP: Maltose-binding protein
mBBr: Monobromobimane
MD: Molecular dynamics simulations
PBPs: Periplasmic binding proteins
Rg: Radius of gyration
RMSD: Root-mean-square deviation
RMSF: Root-mean-square fluctuation
SASA: Solvent-accessible surface area
SDS–PAGE: Sodium dodecyl sulfate–polyacrylamide gel electrophoresis
TrIQ: Tryptophan/tyrosine-induced quenching
