# Supplementary material for: Rational design framework for fluorescent biosensors from periplasmic binding proteins
Source: Biosci Rep. 2026 May 8;46(5):BSR20260131. doi: 10.1042/BSR20260131 (PMC13161334; doi:10.1042/BSR20260131)
Supplement: Supplementary Figures S1-S12 and Table S1 [file BSR-2026-0131_supp.pdf]

Supplementary Information

EXPERIMENTAL FRAMEWORK FOR RATIONAL BIOSENSORS DESIGN FROM PERIPLASMIC BINDING PROTEINS

*Martín González-Andrade[a], Alejandro Sosa-Peinado[a], and Nathaly Vasquez-Martínez[a]**

[a] *Facultad de Medicina, Universidad Nacional Autónoma de México, Ciudad de México, 04510, México.*

Keywords: fluorescent biosensor, rational design, site-specific labeling, periplasmic binding protein, LAO, mBBr

***The corresponding author**: Nathaly Vasquez Martìnez. Email: nathaly@bq.unam.mx

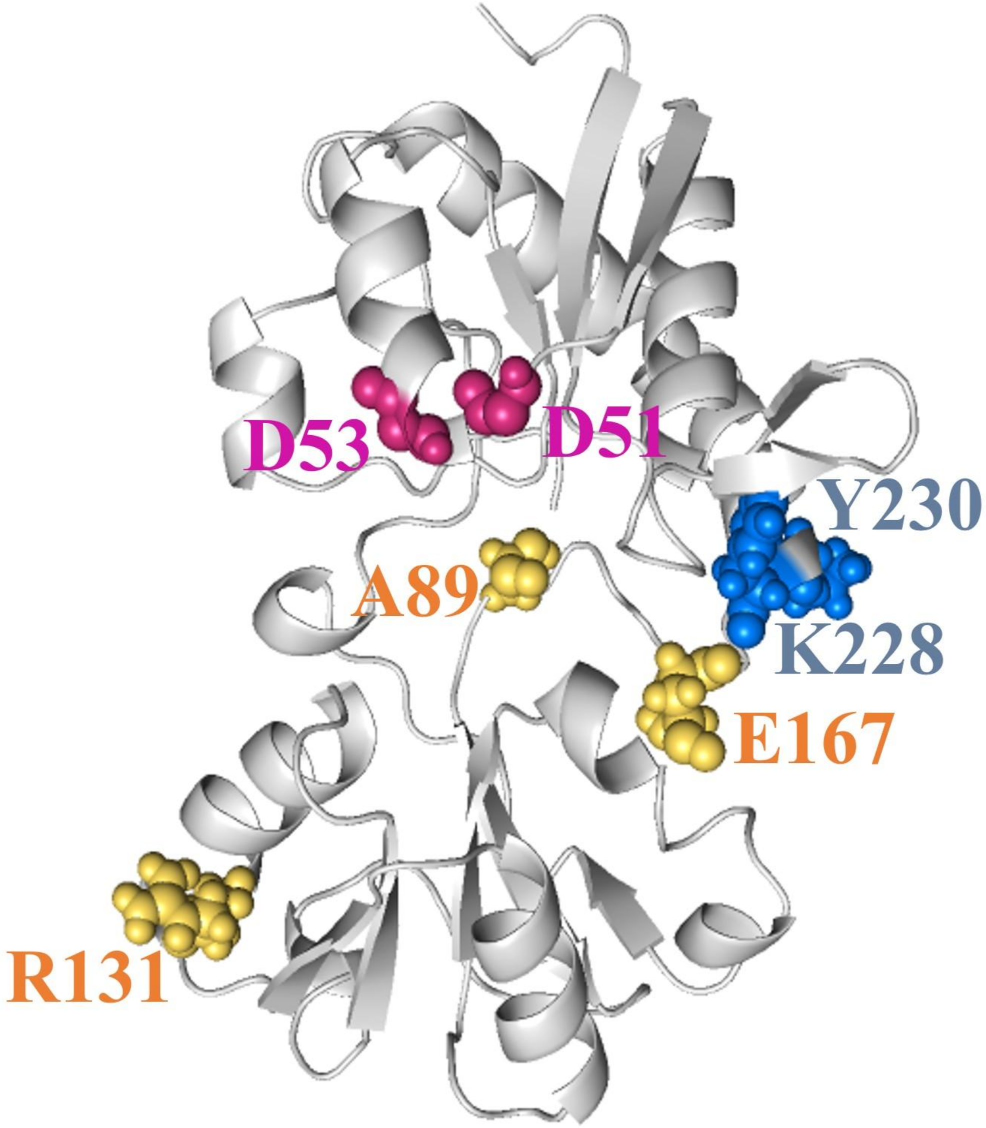

Figure S1. Structural overview of LAO and location of mBBr-labeled positions. A. Crystal structure of LAO in the open, ligand-free conformation (PDB: 2LAO); structural elements: Domain A (cyan), Domain B (yellow), hinge region (orange), and highly flexible flap (pink). C. Location of the seven mBBr-labeled positions on the open LAO structure: endosteric positions D51 and D53 (magenta, spheres), peristeric positions A89, R131, and E167 (yellow, spheres), and allosteric positions K228 and Y230 (blue, spheres).

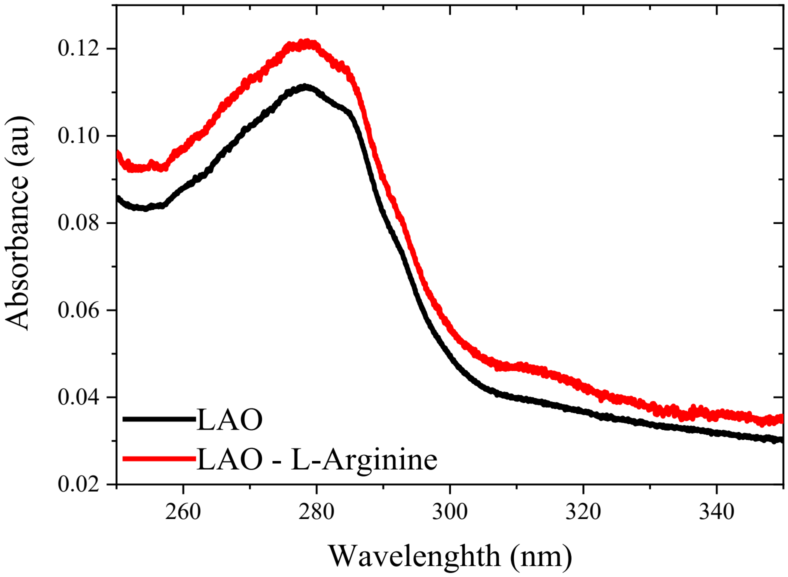

Figure S2. **UV-visible absorption spectra of wild-type LAO.** Apo-LAO (black) and L-arginine-bound LAO (red) show characteristic protein absorption at 278 nm with distinct spectral differences confirming conformational separation.

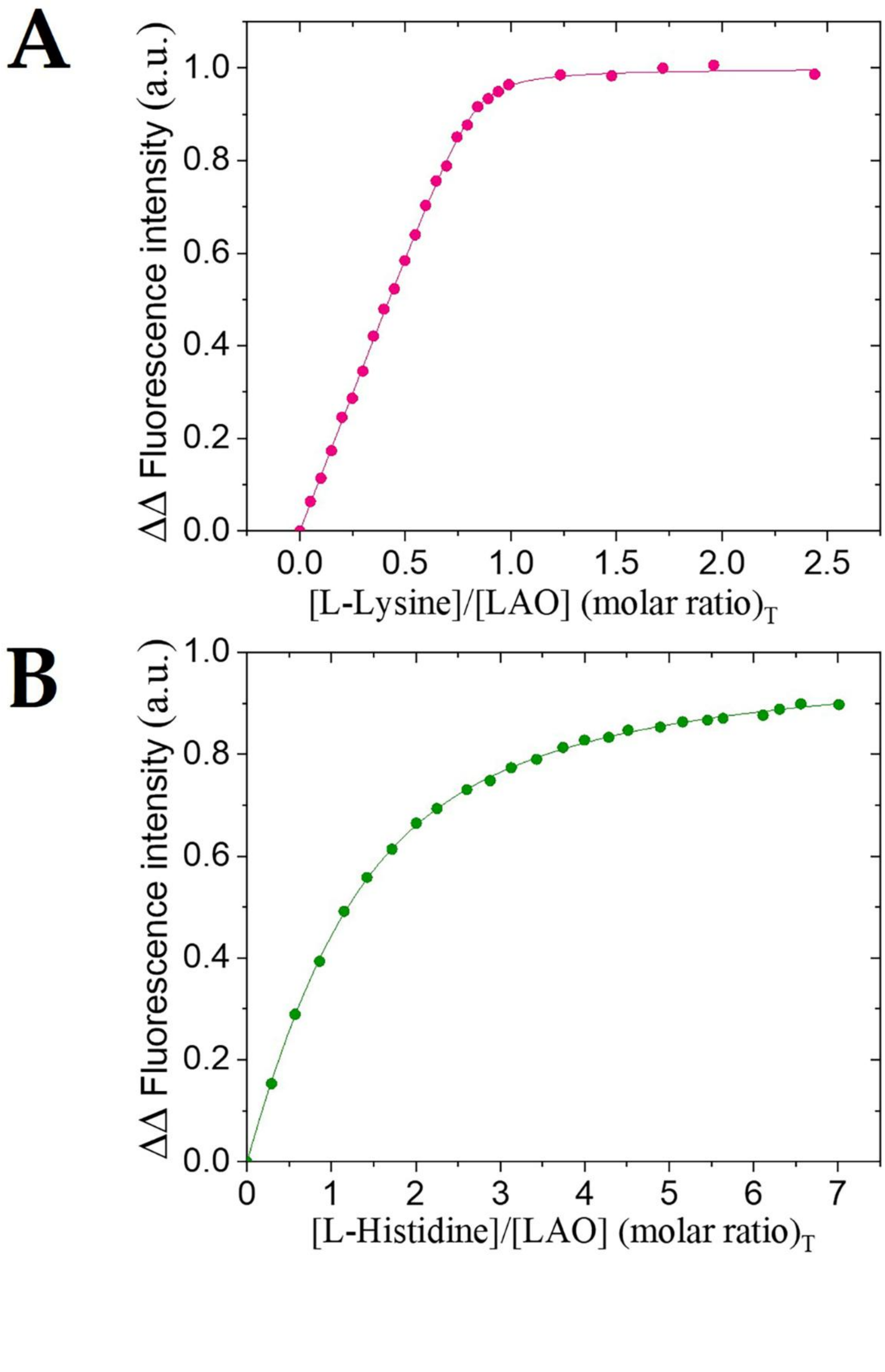

A
ΔΔ Fluorescence intensity (a.u.)
[L-Lysine]/[LAO] (molar ratio)T
B
ΔΔ Fluorescence intensity (a.u.)
[L-Histidine]/[LAO] (molar ratio)T

Figure S3. Ligand binding of wild-type LAO to L-Lysine and L-Histidine. Binding isotherms fit to a 1:1 model for A. L-Lysine (K_d = 8 ± 0.21 nM) and B. L-Histidine (K_d = 687 nM). Fluorescence intensity is expressed in arbitrary units (a.u.).

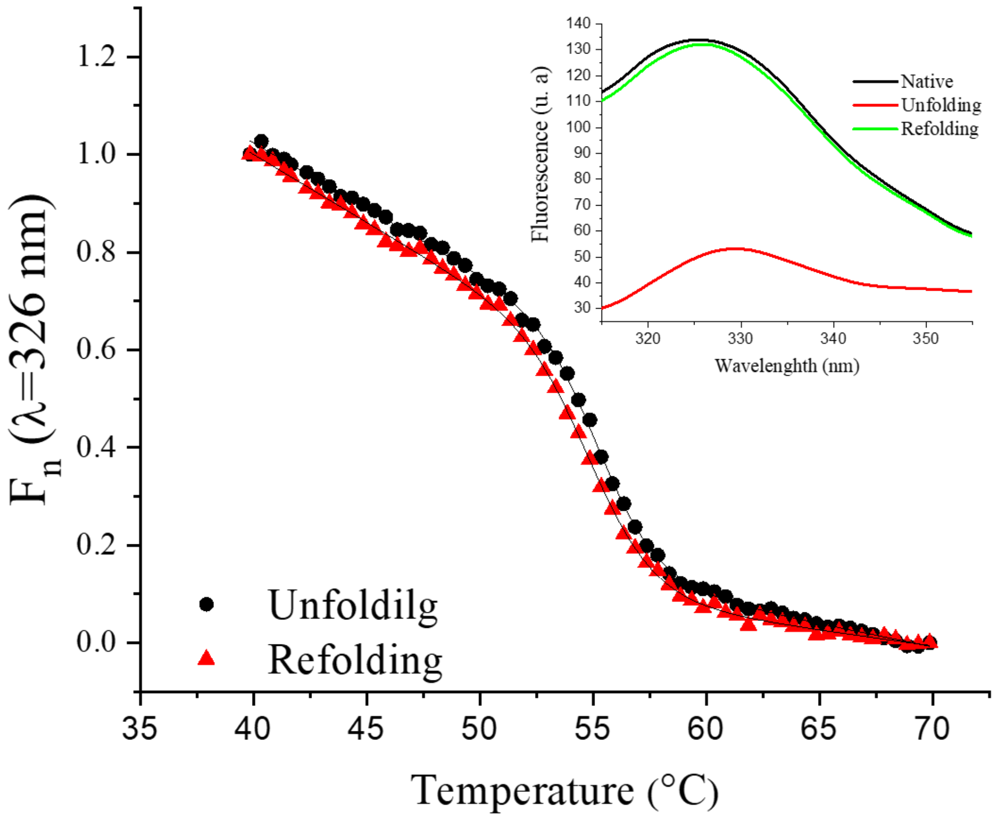

Figure S4. Reversible thermal unfolding of wild-type LAO. Temperature-induced unfolding (black circles) and refolding (red triangles) were monitored by changes in intrinsic tryptophan fluorescence at 326 nm (λ_{ex} = 295 nm). Solid lines show fits to a two-state equilibrium model (Equation 3). Inset: fluorescence emission spectra of native (red), thermally unfolded (black), and refolded (green) protein states, demonstrating reversibility of the thermal transition.

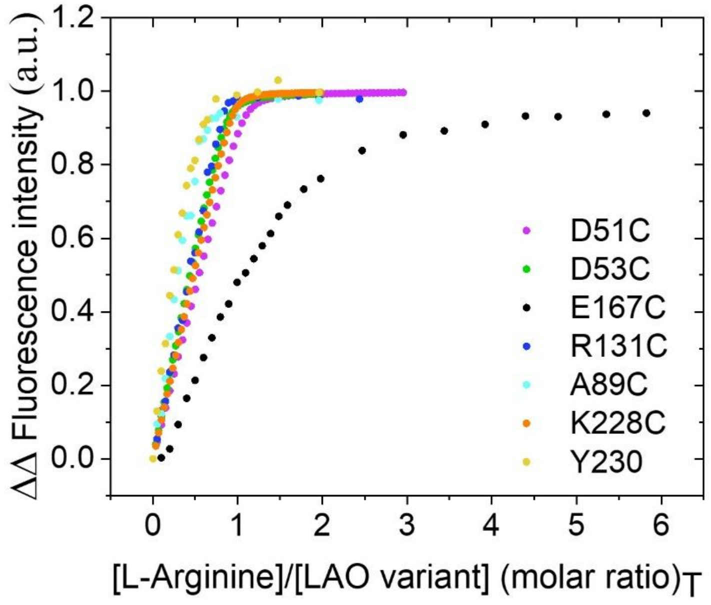

Figure S5. Fluorescence titration curves of mBBr-labeled LAO variants with L-arginine. normalized fluorescence intensity as a function of L-arginine concentration for seven mBBr-labeled variants (1 μM protein, 30°C, λex = 381 nm). Curves are normalized to the initial apo-state intensity. Solid lines: fits to 1:1 binding model.

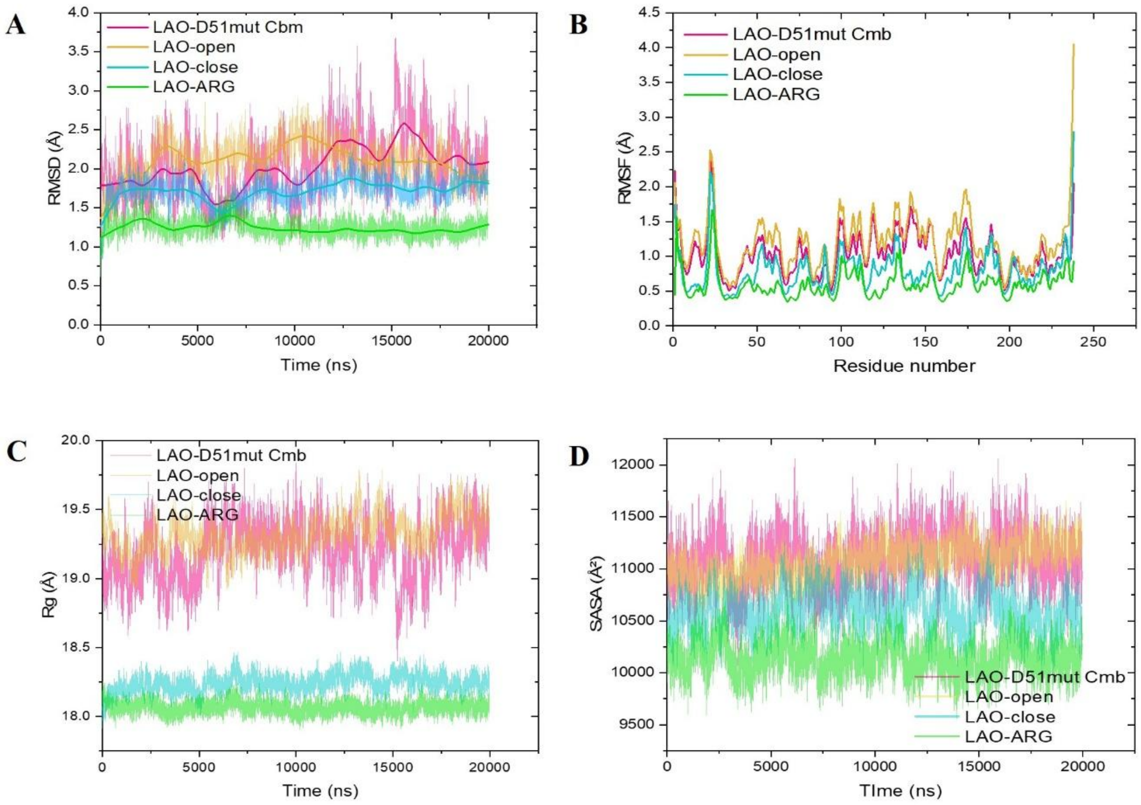

Figure S6. Conformational dynamics of the D51C mBBr-labeled LAO variant during molecular dynamics simulations. Comparative analysis of structural parameters over 200-ns MD trajectories for wild-type LAO in three states (open apo-LAO, yellow; closed apo-LAO, cyan; L-arginine-bound LAO, green) and the D51C mBBr-labeled single-cysteine variant (pink). A. Root-mean-square deviation (RMSD) of backbone atoms calculated against the equilibrated structure; B. Root-mean-square fluctuation (RMSF) per residue number; C. Radius of gyration (Rg); and D. solvent-accessible surface area (SASA).

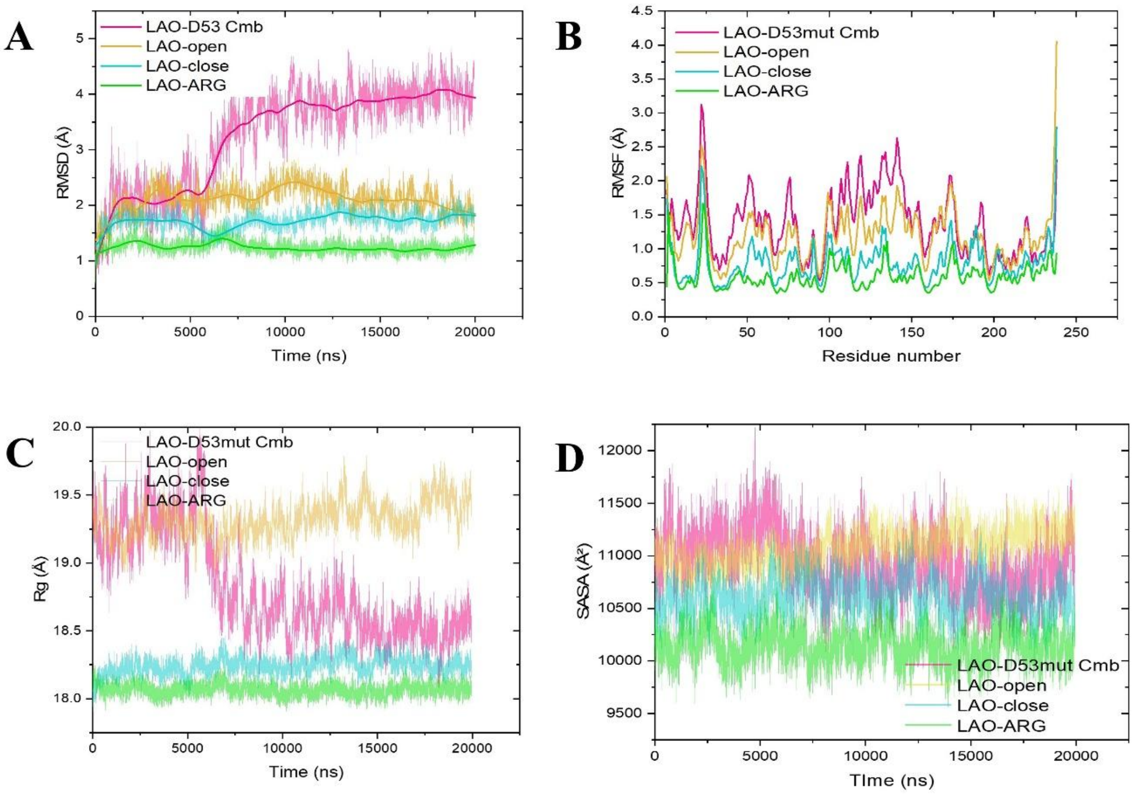

Figure S7. Conformational dynamics of the D53C mBBr-labeled LAO variant during molecular dynamics simulations. Comparative analysis of structural parameters over 200-ns MD trajectories for wild-type LAO in three states (open apo-LAO, yellow; closed apo-LAO, cyan; L-arginine-bound LAO, green) and the D53C mBBr-labeled single-cysteine variant (pink). A. Root-mean-square deviation (RMSD) of backbone atoms calculated against the equilibrated structure; B. Root-mean-square fluctuation (RMSF) per residue number; C. Radius of gyration (Rg); and D. solvent-accessible surface area (SASA).

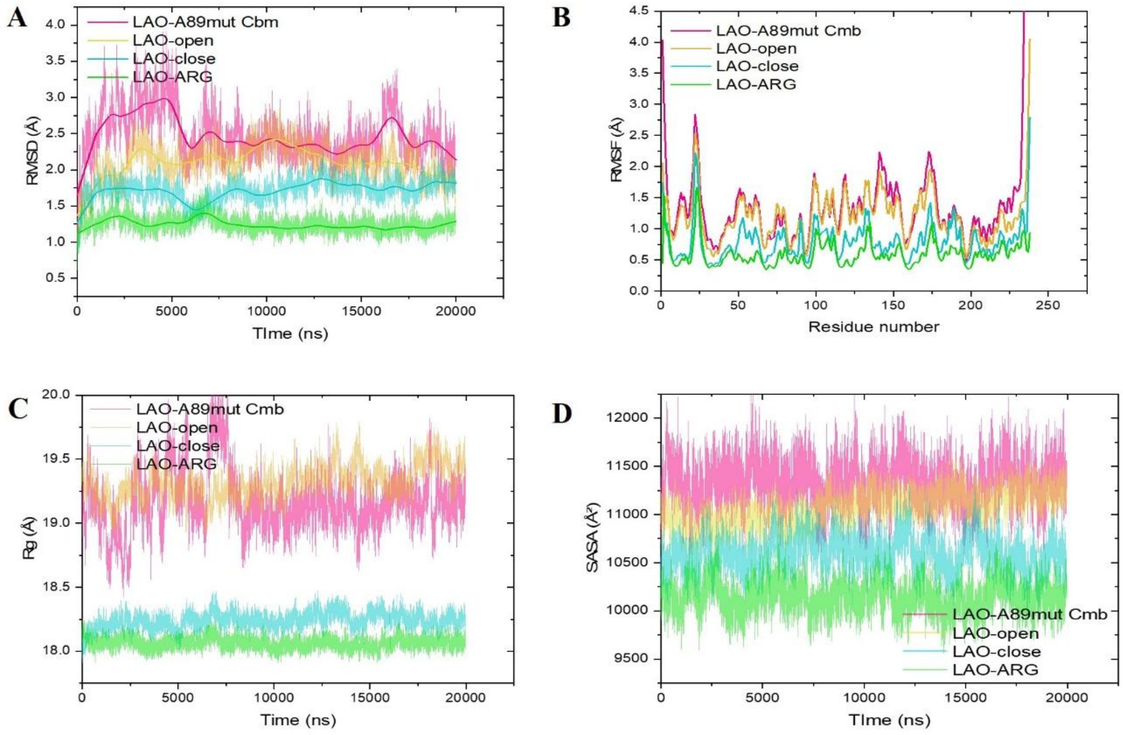

Figure S8. Conformational dynamics of the A89C mBBr-labeled LAO variant during molecular dynamics simulations. Comparative analysis of structural parameters over 200-ns MD trajectories for wild-type LAO in three states (open apo-LAO, yellow; closed apo-LAO, cyan; L-arginine-bound LAO, green) and the A89C mBBr-labeled single-cysteine variant (pink). A. Root-mean-square deviation (RMSD) of backbone atoms calculated against the equilibrated structure; B. Root-mean-square fluctuation (RMSF) per residue number; C. Radius of gyration (Rg); and D. solvent-accessible surface area (SASA).

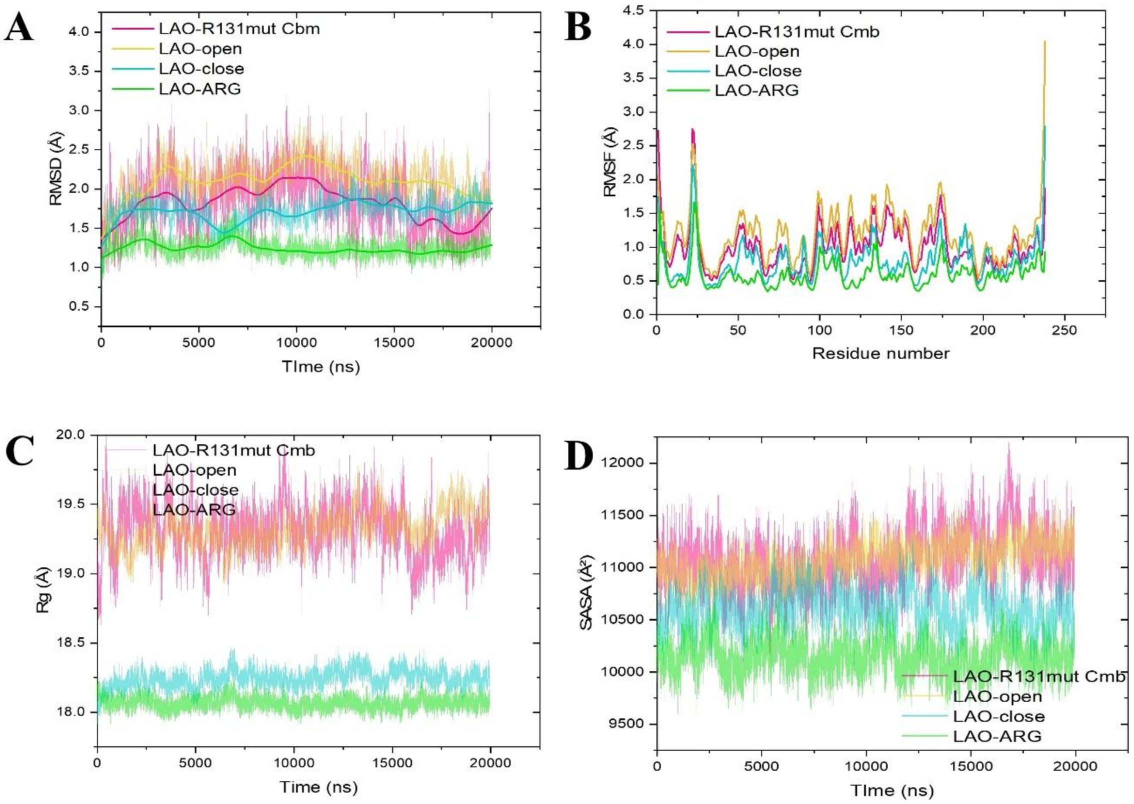

Figure S9. Conformational dynamics of the R131C mBBr-labeled LAO variant during molecular dynamics simulations. Comparative analysis of structural parameters over 200-ns MD trajectories for wild-type LAO in three states (open apo-LAO, yellow; closed apo-LAO, cyan; L-arginine-bound LAO, green) and the R131C mBBr-labeled single-cysteine variant (pink). A. Root-mean-square deviation (RMSD) of backbone atoms calculated against the equilibrated structure; B. Root-mean-square fluctuation (RMSF) per residue number; C. Radius of gyration (Rg); and D. solvent-accessible surface area (SASA).

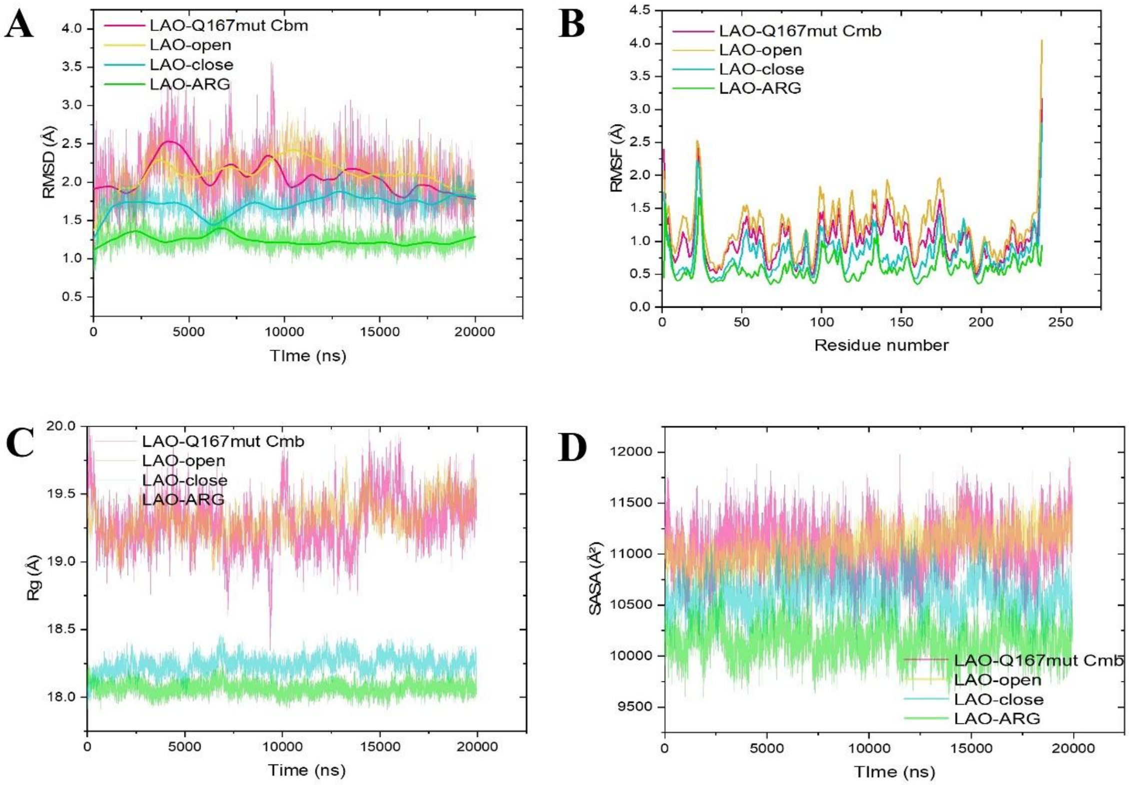

Figure S10. Conformational dynamics of the Q167C mBBr-labeled LAO variant during molecular dynamics simulations. Comparative analysis of structural parameters over 200-ns MD trajectories for wild-type LAO in three states (open apo-LAO, yellow; closed apo-LAO, cyan; L-arginine-bound LAO, green) and the Q167C mBBr-labeled single-cysteine variant (pink). A. Root-mean-square deviation (RMSD) of backbone atoms calculated against the equilibrated structure; B. Root-mean-square fluctuation (RMSF) per residue number; C. Radius of gyration (Rg); and D. solvent-accessible surface area (SASA).

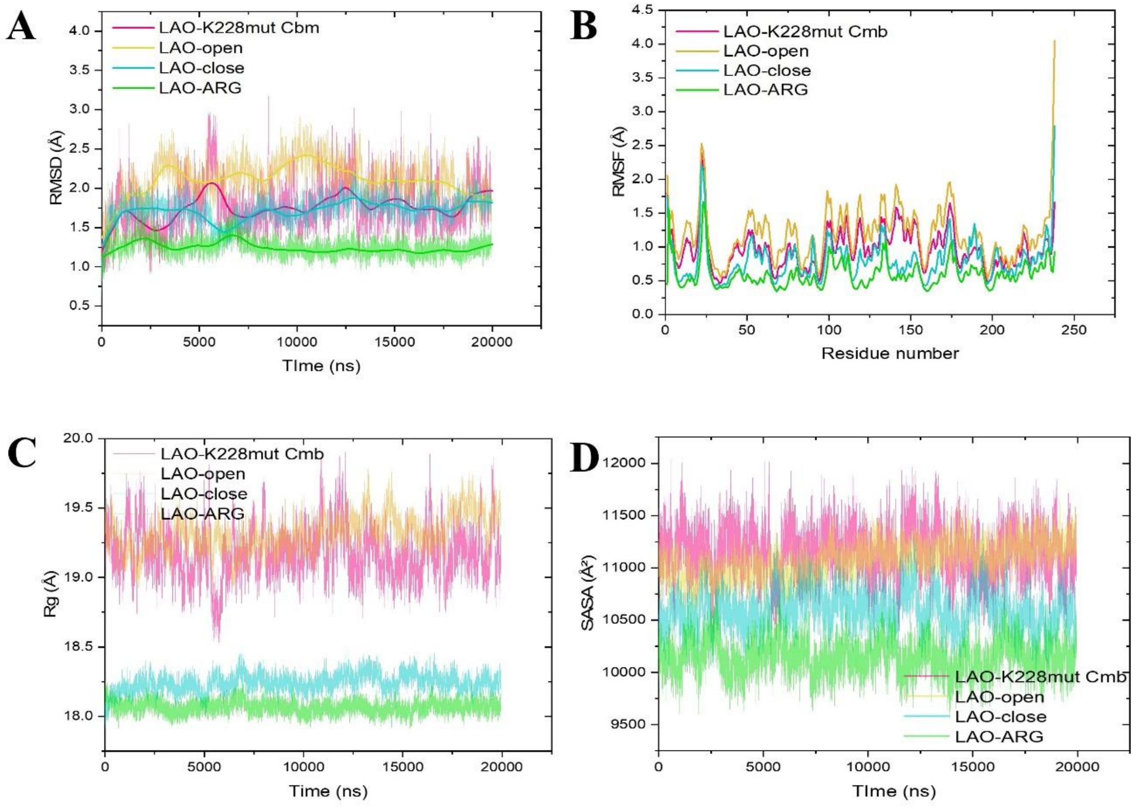

Figure S11. Conformational dynamics of the K228C mBBr-labeled LAO variant during molecular dynamics simulations. Comparative analysis of structural parameters over 200-ns MD trajectories for wild-type LAO in three states (open apo-LAO, yellow; closed apo-LAO, cyan; L-arginine-bound LAO, green) and the K228C mBBr-labeled single-cysteine variant (pink). A. Root-mean-square deviation (RMSD) of backbone atoms calculated against the equilibrated structure; B. Root-mean-square fluctuation (RMSF) per residue number; C. Radius of gyration (Rg); and D. solvent-accessible surface area (SASA).

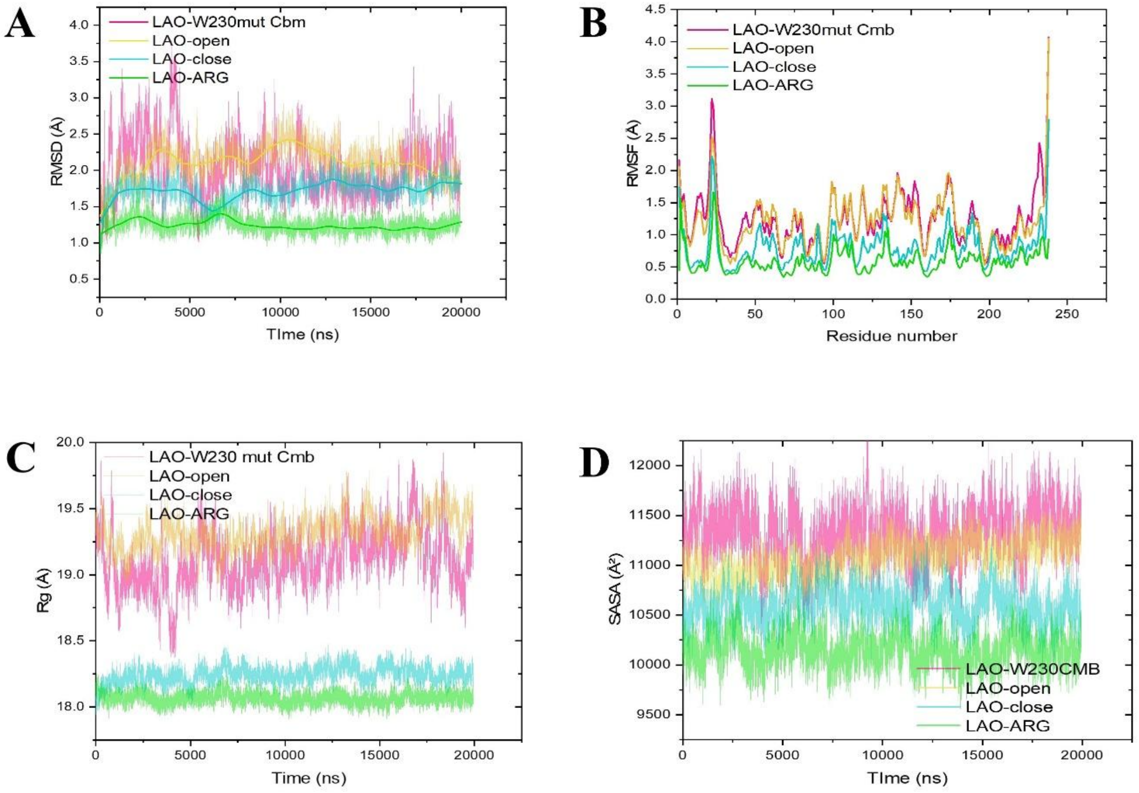

Figure S12. Conformational dynamics of the W230C mBBr-labeled LAO variant during molecular dynamics simulations. Comparative analysis of structural parameters over 200-ns MD trajectories for wild-type LAO in three states (open apo-LAO, yellow; closed apo-LAO, cyan; L-arginine-bound LAO, green) and the W230C mBBr-labeled single-cysteine variant (pink). A. Root-mean-square deviation (RMSD) of backbone atoms calculated against the equilibrated structure; B. Root-mean-square fluctuation (RMSF) per residue number; C. Radius of gyration (Rg); and D. solvent-accessible surface area (SASA).

**Table S1. Proximity analysis of mBBr labeling positions to potential TrIQ quenching residues**					
**Variant**	**Aromatic residue**	**Residue number**	**Residue type**	**Distance (Å)**	**Potential quenching†**
D51C	Y14	14	Tyr	11.64	yes
D51C	W47	47	Trp	14.33	yes
D51C	Y73	73	Tyr	20.4	no
D51C	Y126	126	Tyr	28.31	no
D51C	W130	130	Trp	33.12	no
D51C	Y142	142	Tyr	22.28	no
D51C	Y218	218	Tyr	30.16	no
D53C	Y14	14	Tyr	13.41	yes
D53C	W47	47	Trp	18.05	no
D53C	Y73	73	Tyr	17.78	no
D53C	Y126	126	Tyr	24.91	no
D53C	W130	130	Trp	29.61	no
D53C	Y142	142	Tyr	21.26	no
D53C	Y218	218	Tyr	30.38	no
A89C	Y14	14	Tyr	15.41	no
A89C	W47	47	Trp	23.17	no
A89C	Y73	73	Tyr	10.44	yes
A89C	Y126	126	Tyr	18.65	no
A89C	W130	130	Trp	20.8	no
A89C	Y142	142	Tyr	25.89	no
A89C	Y218	218	Tyr	13.88	yes
R131C	Y14	14	Tyr	28.7	no
R131C	W47	47	Trp	41.02	no
R131C	Y73	73	Tyr	25.98	no
R131C	Y126	126	Tyr	5.96	yes
R131C	W130	130	Trp	5.22	yes
R131C	Y142	142	Tyr	19.43	no
R131C	Y218	218	Tyr	37.95	no
E167C	Y14	14	Tyr	10.01	yes
E167C	W47	47	Trp	21.69	no
E167C	Y73	73	Tyr	21.47	no
E167C	Y126	126	Tyr	21.39	no
E167C	W130	130	Trp	24.89	no
E167C	Y142	142	Tyr	18.08	no
E167C	Y218	218	Tyr	23.08	no
K228C	Y14	14	Tyr	19.51	no
K228C	W47	47	Trp	22.47	no
K228C	Y73	73	Tyr	24.18	no
K228C	Y126	126	Tyr	32.24	no
K228C	W130	130	Trp	34.45	no
K228C	Y142	142	Tyr	33.43	no

**Table S1. Proximity analysis of mBBr labeling positions to potential TrIQ quenching residues**					
**Variant**	**Aromatic residue**	**Residue number**	**Residue type**	**Distance (Å)**	**Potential quenching†**
K228C	Y218	218	Tyr	13.82	yes
Y230C	Y14	14	Tyr	12.04	yes
Y230C	W47	47	Trp	17.98	no
Y230C	Y73	73	Tyr	20.55	no
Y230C	Y126	126	Tyr	27.32	no
Y230C	W130	130	Trp	30.37	no
Y230C	Y142	142	Tyr	26.38	no
Y230C	Y218	218	Tyr	16.33	no
Distances measured between Cβ of labeling position and aromatic ring center in open LAO (PDB: 2LAO). Potential quenching: yes, distance <15 Å; no, distance ≥15 Å.					
